# Elucidating the Role of Lignin Type and Functionality in the Development of High-Performance Biobased Phenolic Thermoset Resins

**DOI:** 10.1021/acsapm.3c02136

**Published:** 2024-01-10

**Authors:** Emanuela Bellinetto, Nicholas Fumagalli, Matilde Astorri, Stefano Turri, Gianmarco Griffini

**Affiliations:** Department of Chemistry, Materials and Chemical Engineering “Giulio Natta”, Politecnico di Milano, Piazza Leonoardo da Vinci 32, 20133 Milano, Italy

**Keywords:** lignin, phenolic resins, biobased materials, lignocellulosic biomass, biobased thermosets

## Abstract

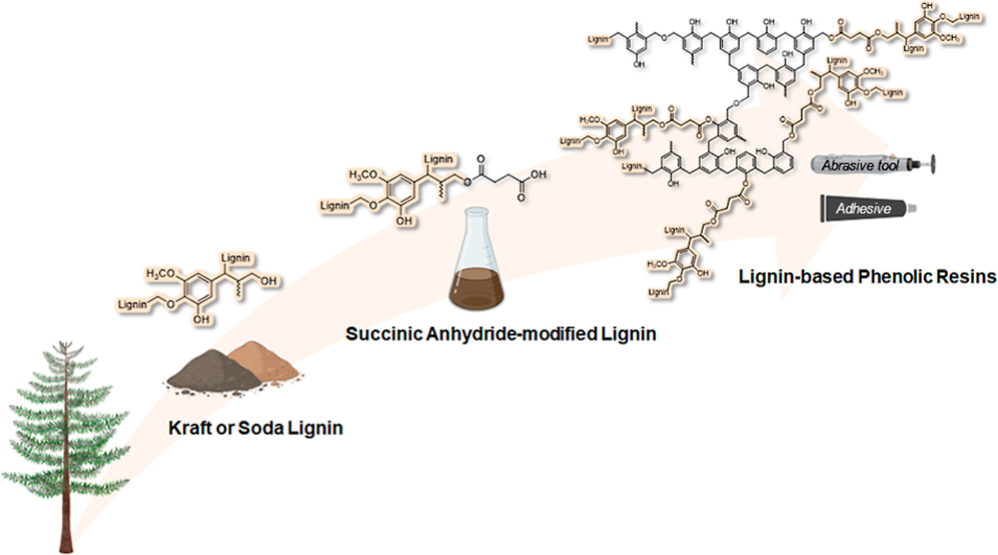

In this work, a series of biobased phenolic resins were developed starting from kraft and soda lignin, suitably functionalized through esterification by means of succinic anhydride. As a result of an extensive optimization study of the functionalization and curing reactions, clear correlations between lignin type and chemical–physical characteristics and the properties of the resulting phenolic resin systems were described. In particular, the esterification reaction through succinic anhydride was found to play a key role in enhancing the chemical reactivity and in facilitating the successful incorporation of lignin into the resin formulations. The obtained high-lignin-content thermoset materials were shown to exhibit tunable chemical (functionality, gel content, and cross-linking density), thermal (glass transition temperature and thermo-oxidative stability), and mechanical (surface hardness, indentation modulus, and creep behavior) characteristics, which could outperform those of fully oil-based reference phenolic resins by judicious control of lignin concentration and chemical characteristics. In particular, succinylated kraft lignin was found to enable more efficient incorporation into the cured systems. This work provides the first demonstration of the incorporation of succinic-anhydride-modified-lignin in the formulation of high-performance phenolic resins, ultimately contributing to the definition of structure–property–performance correlations for rational biobased material design in the context of advanced and sustainable manufacturing.

## 1 Introduction

Phenolic resins, obtained by the reaction between phenol and formaldehyde (PF), are currently among the most widely used thermosetting materials globally, due to their excellent properties and relatively low production cost.^1^ PF resins are characterized by high cross-linking density and high content of aromatic rings, which are responsible for their superior thermal, chemical, and mechanical stability, as well as moisture resistance and good mechanical strength. For these reasons, their fields of application are manifold and include wood composites, isolating materials, adhesives, and binders.^2^

Since PF resins are obtained by polycondensation of phenol and formaldehyde, which are both fossil-based and toxic precursors, the need of employing more environmentally friendly, renewable, and less hazardous raw materials for their production has recently emerged.^3,4^

In this context, thanks to its natural polyphenolic structure, lignin can be regarded as a promising alternative to oil-derived phenols,^5^ whose vapors are harmful to human eyes, skin, respiratory apparatus, central nervous system, and heart.^6^

Lignin is the most abundant natural aromatic polymer on earth, constituting between 17 and 33% of the mass of trees and plants.^7^ It is largely available as a byproduct of the pulp and paper industry, with an annual production exceeding 50 million of tons, of which only 2% is used in value-added products.^8^ In particular, the current limitations in lignin exploitation within the polymer manufacturing sector are due to its large molecular weight and broad molecular weight distribution, its structural variability and complexity, together with the presence of few reactive sites.^9^ Moreover, structural characteristics of technical lignins change according to the extraction method adopted (i.e., sulfite, kraft, organosolv, or soda).^10^ Among these, soda and kraft processes are of particular interest for high-value applications as they are the most widely used pulping technologies, providing high yields and highly pure technical lignins,^11^ generally richer in phenolic hydroxyls.^10^

Different studies have recently focused on lignin modification, with the aim of increasing its reactivity toward PF systems mainly through chemical approaches, by modifying lignin particles size^12,13^ or functionalities.^14^ In the latter strategies, hydroxymethylation,^15,16^ phenolation,^17,18^ and demethylation,^19,20^ in some cases in combination with depolymerization and/or fractionation, have led to significant results, particularly in the context of PF adhesives.^14,21,22^

For instance, Lee et al. used phenolated-hydroxymethylated organosolv and soda lignin as a partial substituent of phenol in a phenolic adhesive formulation. The lignin-modified resins exhibited lower thermal stability but comparable bonding strength to that of reference PF resins when substitution occurs with 10 wt % of organosolv lignin and 5 wt % of soda lignin.^23^ In other works, kraft lignin and different lignosulfonates were subjected to phenolation and solvent fractionation. As a result of the chemical modification, 40 wt % of kraft lignin substitution to phenols provided a bonding strength comparable to that of standard PF adhesives. Conversely, the performance of lignosulfonate-based resins was always found to be lower than that of the reference material.^24,25^ To the same end, demethylation of soda lignin performed by Wang et al. led to higher bonding performance of the resin if compared to pristine lignin-modified thermoset, but at the expense of lower thermal stability, lower glass transition temperature and lower bond strength with respect to the reference adhesive.^26^

Examples of lignin-modified PF resins have also been proposed for use as binders in the production of abrasive tools.^27,28^ Among these, Klapiszewski et al. investigated the possibility to employ magnesium lignosulfonate and kraft lignin after their activation by oxidation reaction. According to dynamic mechanical analysis results, the model composites containing 80% of abrasive grains, 12% of novolac (with 9% hexamine), 3% of resole, and 5% of magnesium lignosulfonate oxidized by H_2_O_2_ showed the best thermomechanical characteristics (i.e., higher storage modulus at 25 °C).^29^

Despite the viability of such approaches for increasing lignin reactivity in PF systems, some major drawbacks are still present. Indeed, upon functionalization, additional hazardous phenols and/or formaldehyde are typically required for lignin phenolation and hydroxymethylation, while demethylation often needs high temperatures and pressures, restricting its wide-scale applicability.^14,22^ Furthermore, the reaction media used for most oxidation reactions are highly alkaline solutions. This necessarily involves a secondary step to isolate the reaction products, usually by acidification of the reaction medium and extraction of the product with an organic solvent, thus requiring large amounts of acid in addition to solvents.^30^

A different approach aimed at lignin functionalization is esterification. Esterification by means of different carboxylic diacids and anhydrides has been successfully used in some recent works to enhance lignin reactivity through the incorporation of more accessible pendant carboxylic moieties, covalently attached to the lignin macromolecule.^31,32^ In particular, lignin esterification by succinic anhydride (SAn) was effectively used in a previous work from our group to prepare lignin-based cross-linked polyester coatings.^33^ This functionalization route represents a very promising way in the contest of PF resins, considering the acceleration effect associated with the presence of ester groups in curing reactions between phenols and formaldehyde.^34^ Additionally, the functionalizing agent used (i.e., SAn) can be bioderived from the dehydration of succinic acid.^35^ However, very little work has been done on the exploitation of this functionalization approach for the modification of lignin and its application in PF systems, despite its great potential.^36^

Based on the considerations above, an esterification process using SAn as a functionalizing agent was employed in this work to enhance lignin reactivity in the context of PF resins. The obtained succinylated lignins were used as a replacement of the novolac precursor in commercial PF formulations to obtain high-lignin-content high-performance biobased thermoset materials. To elucidate the effect of lignin type and chemical–physical characteristics (viz., lignin extraction method) on the properties of the phenolic resinoid system, the functionalization process and the subsequent reactions to obtain the final thermosets were studied using both soda and kraft lignins. The benchmark resin and the high-lignin-content materials were extensively characterized with respect to their curing activation energy, gel content, glass transition temperature (*T*_g_), thermo-oxidative stability, cross-linking density, and hardness, ultimately providing useful structure–property-performance correlations for rational biobased material design.

## 2 Experimental Section

### 2.1 Materials

Softwood kraft lignin (KL) Indulin AT was supplied by Ingevity, and soda lignin (SL) Protobind 1000 was purchased from Tanovis AG. SL (average particle size 1–10 μm) was used as received, while KL (average particle size 10–100 μm) was subjected to ball milling prior to use in order to achieve for both lignins similar granulometry [scanning electron microscopy (SEM) images are reported in Figure S1 in the Supporting Information].

For the functionalization reaction of both lignins, SAn, 1-methylimidazole (1-MI) and tetrahydrofuran (THF) were used as received from Sigma-Aldrich. The commercial PF resin formulation employed was provided by Camfart S.r.l. and was composed by the novolac Bakelite PF SM 1112 [containing phenols and 8.5–9.5 wt % hexamethylenetetramine (HMTA), in powder form] and the liquid resole Bakelite PF 2770, consisting of phenols and <1 wt % of free formaldehyde.

### 2.2 Lignin Functionalization

**Figure 1:**
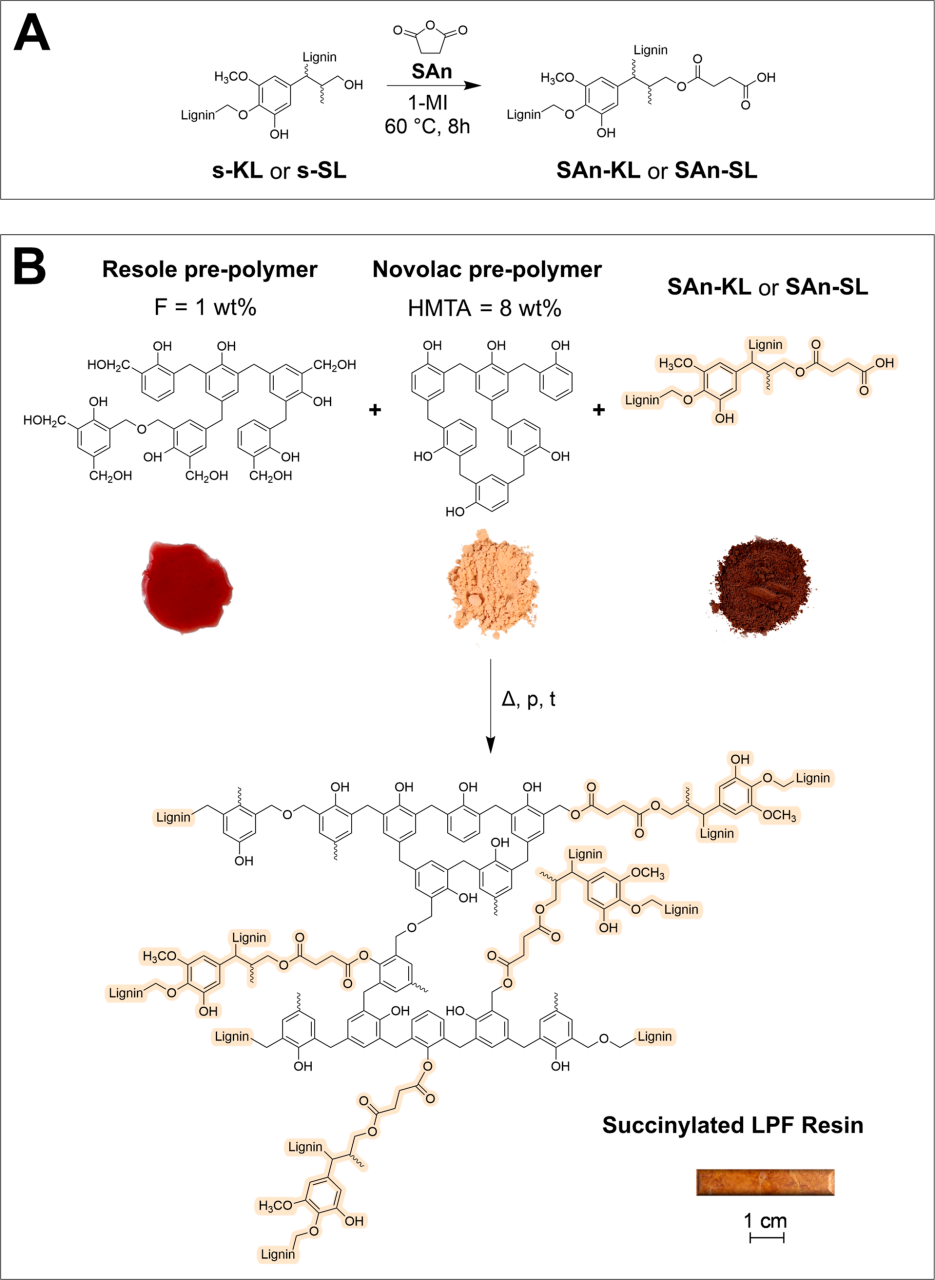
(A) Functionalization reaction of s-KL/s-SL with SAn leading to SAn-KL/SAn-SL. (B) Possible reactions between SAn-lignin, resole, and novolac prepolymer in the synthesis of succinylated LPF-resins. For illustrative purposes, a slab of succinylated LPF resin (LPF SAn-KL 5) has been reported.

For the esterification reaction, THF-soluble fractions of KL (s-KL) or SL (s-SL) lignins (obtained via Soxhlet extraction, 24 h at 85 °C; 4 g of lignin per 150 mL of THF) and SAn (SAn/lignin aliphatic OH molar ratio = 2) were dissolved in THF in the presence of 1-MI as the catalyst (0.2 mL/g of s-KL or s-SL) in a three-neck round-bottom flask and allowed to vigorously stir for 8 h at 60 °C in a nitrogen atmosphere, following a procedure recently reported by our group.^33^ The completion of the reaction was assessed by means of quantitative nuclear magnetic resonance (^31^P NMR) and Fourier transform infrared (FTIR) spectroscopy. At the end of the reaction, the solvent was removed by rotary evaporation and the reaction products were washed twice with distilled water to remove the unreacted anhydride and then, dried under vacuum for 24 h. A schematic representation of the functionalization process is shown in Figure 1A.

### 2.3 Lignin-Based PF Resins Production

Lignin-based PF (LPF) resins were produced employing 15 wt % of resole and 85 wt % of a combination of novolac prepolymer with pristine or succinylated lignins (SAn-KL or SAn-SL) in 100/0, 95/5, 90/10, 80/20, 70/30, and 60/40 novolac/lignin weight ratios. Lignin was manually premixed with the novolac component. Once the powdery phase appeared homogeneous by visual inspection, the resole component was added and mixed with the powder in an aluminum ramekin. The resinoid blends thus obtained were then analyzed by means of DSC measurements (see Section 2.4) in order to monitor their behavior upon heating to determine their activation energy for curing, and to design the optimal curing process to obtain the final lignin-based materials. According to the results obtained from such optimization study, all resins were cured in a hydraulic press (50 bar) in a 10 cm × 10 cm mold, keeping the temperature at 85 °C for 15 min, then at 150 °C for 30 min, and finally at 180 °C for 15 min. A representation of the possible reactions occurring between the resole, the novolac prepolymer, and succinylated lignins during the curing steps is provided in Figure 1B.

### 2.4 Materials Characterization

FTIR analyses were performed by a Nicolet Nexus 760 FT-IR spectrophotometer to study the chemical changes that occurred in lignin after Soxhlet extraction and for monitoring the esterification reaction with SAn. Samples to be analyzed were prepared by pressing lignin with KBr powder to obtain thin discs or by depositing a thin layer of reactants on a KBr disc, then allowing the solvent to evaporate. FTIR was also used to assess the chemical modifications occurring in the resins before and after the application of the curing cycle. In this case, the cured resins were finely ground and then pressed with a KBr powder. Spectra were registered in the transmission mode, at room temperature, in air, by recording 64 accumulated scans at a resolution of 4 cm^–1^ in the 4000–600 cm^–1^ wavenumber range. All of the obtained FTIR spectra were normalized considering the signal at 1515 cm^–1^ (related to pure aromatic skeletal vibrations in lignin) as the invariant band.

Quantitative ^31^P NMR spectroscopy was used to chemically characterize the lignin materials and assess the type and abundance of hydroxyl and carboxyl functionalities. Spectra were recorded by inverse gate proton decoupling sequences on a Bruker Avance 500 spectrometer with 5 mm direct detection broadband probe-head at 27 °C with 384 transients using 90° pulse flip angle, 1 s acquisition time, and 5 s relaxation delay. Pristine lignins, lignin THF-soluble fractions, and succinylated lignins were prepared for the analysis according to a procedure described in the literature.^37^ The integration regions were as follows: 152.8–152.5 ppm (internal standard); 150.0–145.0 ppm (aliphatic hydroxyls); 145.0–137.0 ppm (phenolic hydroxyls); and 137.0–134.5 ppm (carboxylic groups).

The curing kinetics of the resins was studied by applying the Ozawa and the Kissinger model.^38,39^ To this end, nonisothermal DSC analyses were carried out on the uncured lignin-resin blends at different heating rates (β = 5, 10, and 20 °C/min), from room temperature to 300 °C, to detect the temperature of the exothermal peak (*T*_p_) associated with the cross-linking reaction. The apparent activation energy (*E*_a_) of the system was determined according to eq 1 (Ozawa model) and eq 2 (Kissinger model)
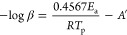
1
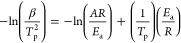
2where *R* is the gas constant and *A*′ and *A* are the Ozawa and Kissinger preindex factors, respectively.

After the curing procedure, the amount of cross-linked material in all the resins produced was assessed by solvent resistance (gel content) tests. The cured samples (*W*_S_ ≈ 1 g) were submerged in THF (a good solvent for lignin, novolac, and resole) for 24 h under stirring and then dried in a vacuum oven at 60 °C for 72 h to completely remove the solvent. Samples were weighed until a constant recovered mass (*W*_D_) was recorded and the solid extracted fraction was calculated as reported in eq 3
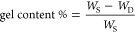
3

DSC analyses were also employed to determine the *T*_g_ values of the resins. Measurements were carried out on 10–15 mg samples by means of a Mettler-Toledo DSC 823e instrument, at a scan rate of 20 °C/min from 25 to 300 °C, under nitrogen flux.

Thermogravimetric analysis (TGA) was performed on the resin samples (∼15 mg) to study their thermal stability using a Q500 TGA system (TA Instruments) from ambient temperature to 800 °C, at a scan rate of 10 °C/min, in air.

TGA/FTIR experiments were carried out from ambient temperature to 800 °C at 10 °C/min in an air atmosphere by coupling the TGA apparatus with a ThermoScientific Nicolet iS50 FTIR spectrometer. FTIR spectra were recorded in the 4000–500 cm^–1^ spectral range with a 4 cm^–1^ resolution, collecting scans every 20 s.

Dynamic mechanical analysis (DMA) was carried out in tension mode, by means of a Mettler Toledo DMA/SDTA 861e dynamic mechanical analyzer to determine the low-strain mechanical response and the cross-linking density of the cured resins. Rectangular specimens with a thickness of around 1 mm and a width of 7 mm were tested in displacement-controlled oscillation, with an amplitude equal to 10 μm and a frequency of 1 Hz, at a clamping distance of 10.5 mm. The storage modulus (*E*′) was monitored over the 25–280 °C temperature range at a heating rate of 3 °C/min. The cross-linking density ν (corresponding to the number of moles of cross-linking sites per unit volume, mol/cm^3^) was obtained according to the rubber elasticity theory, as described by eq 4
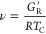
4where *T*_C_ is the characteristic temperature and  is the shear storage modulus in the rubbery plateau at *T*_C_. In this case, the value of *T*_C_ was fixed at 50 °C above the *T*_g_ of the sample under investigation.  is correlated to the tensile storage modulus in the rubbery plateau  according to eq 5
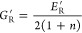
5in which n is the Poisson’s ratio (0.5 for elastically deformed incompressible isotropic materials).

Microindentation tests were carried out on the cured samples to determine both their Vickers surface hardness—*H*_IT_ and their time-dependent response, namely the amount of elastic work done by microindenting the sample—η_IT_, the indentation creep—*C*_IT,1_, and the recovery creep—*C*_IT,2_ (see Figure S9 in the Supporting Information for additional details on the determination of these parameters). Tests were carried out on 2 mm thick samples using a Fischer Scope HP100 V microindenter, equipped with a standard Vickers diamond tip, in controlled-force mode, loading the samples at 5 mN for 30 s, maintaining the force at 5 mN for 30 s, and then releasing the force at the same rate, while the test force, the corresponding indentation depth, and the test time were recorded. Six measurements were carried out for each material in different regions of the samples, and the average and standard deviation were then computed.

## 3 Results and Discussion

### 3.1 Characterization of Modified Lignins

In view of their incorporation in the target PF formulations, all lignin systems were thoroughly analyzed in terms of their chemical–physical characteristics. In particular, pristine KL and SL, their extracted soluble fractions s-KL and s-SL, and the succinylated materials SAn-KL and SAn-SL were subjected to FTIR and ^31^P NMR spectroscopy, and to GPC and DSC analysis.

More specifically, to assess the effect of solvent extraction on the properties of the resulting lignins and to evaluate the effectiveness of the esterification reaction, the abundance of hydroxyl and carboxyl functionalities in the different lignin materials was gauged by means of ^31^P NMR (spectra are reported in the Supporting Information). As shown in Table 1, where the molar concentration of functional groups in the studied materials is reported for pristine, extracted, and functionalized soda and kraft lignins, the Soxhlet extraction process was found to lead to a general reduction of OH functionalities with respect to the pristine material. In particular, aliphatic –OH groups were shown to decrease from 2.5 mmol/g in KL to 1.5 mmol/g in s-KL, and from 2.2 mmol/g in SL to 1.4 mmol/g in s-SL. As discussed in our previous study on kraft lignin,^40^ this behavior may be explained considering the presence of strong intra-/intermolecular hydrogen bonding interactions between hydroxyl groups in lignin, which limit its affinity with the extraction solvent. Most importantly, ^31^P NMR analysis proved that the functionalization of s-KL and s-SL with SAn effectively led to an increase of lignin –COOH functionalities (from 0.4 to 2.9 mmol/g in s-KL and from 0.8 to 2.4 mmol/g in SAn-SL), with a consequent reduction in the abundance of aliphatic –OH groups. This is consistent with recent literature reports, in which aliphatic hydroxyls were found to be more prone to react compared to phenolic hydroxyls in esterification reactions.^41^

**Table 1:** Concentration of Reactive Functional Groups in Pristine, THF-Soluble Fractions and Succinylated Lignins, as Obtained by Means of ^31^P-NMR

sample	aliphatic OH (mmol/g)	aromatic OH (mmol/g)	COOH (mmol/g)
KL	2.5 ± 0.3	4.3 ± 0.3	0.5 ± 0.1
s-KL	1.5 ± 0.3	4.0 ± 0.3	0.4 ± 0.1
SAn-KL	0.4 ± 0.1	4.3 ± 0.2	2.9 ± 0.3
SL	2.2 ± 0.3	3.0 ± 0.2	1.0 ± 0.1
s-SL	1.4 ± 0.2	2.9 ± 0.2	0.8 ± 0.2
SAn-SL	0.5 ± 0.2	3.1 ± 0.3	2.4 ± 0.2

**Figure 2:**
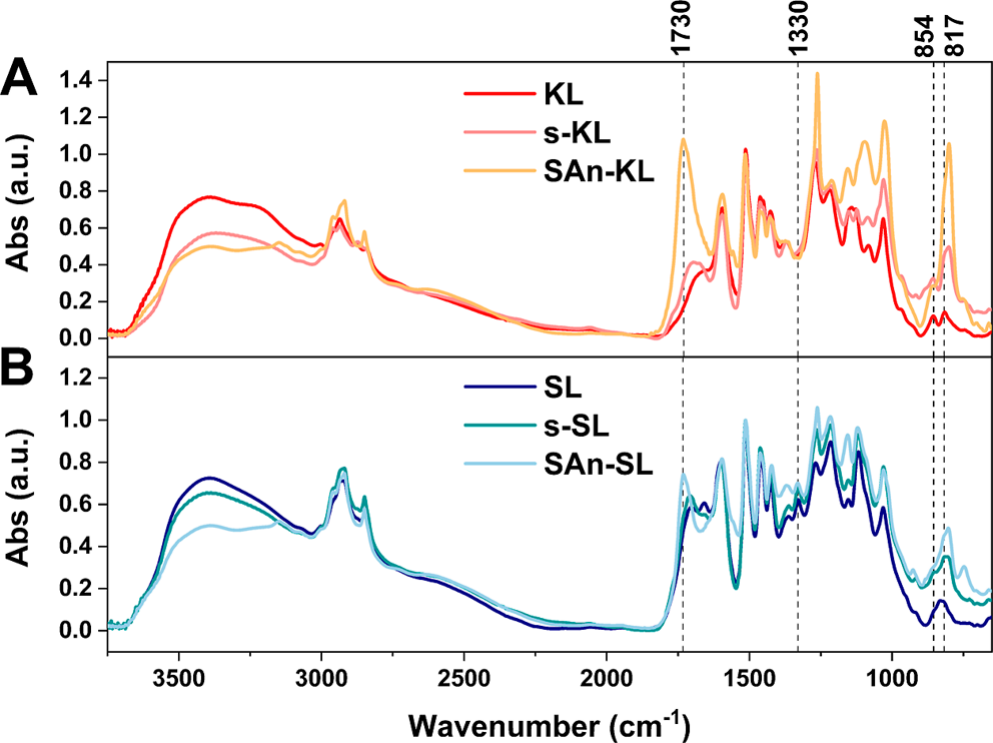
FTIR spectra of pristine, Soxhlet-extracted fractions and succinylated (A) kraft lignin and (B) soda lignin.

These results are also coherent with FTIR analysis (Figure 2), which showed a slightly reduction in the intensity of the –OH stretching signal in the region 3000–3400 cm^–1^ in extracted lignins (both kraft and soda) as compared with their parent materials. Similarly, a broadening and further decrease in –OH signal intensity was found for SAn-KL and SAn-SL with respect to s-KL and s-SL, suggesting a reduction of the hydroxyl group concentration as a consequence of the reaction with SAn. Moreover, a new band appeared at 1730 cm^–1^, attributable to the stretching vibrations of C=O carboxylic groups formed upon esterification.^33^

**Figure 3:**
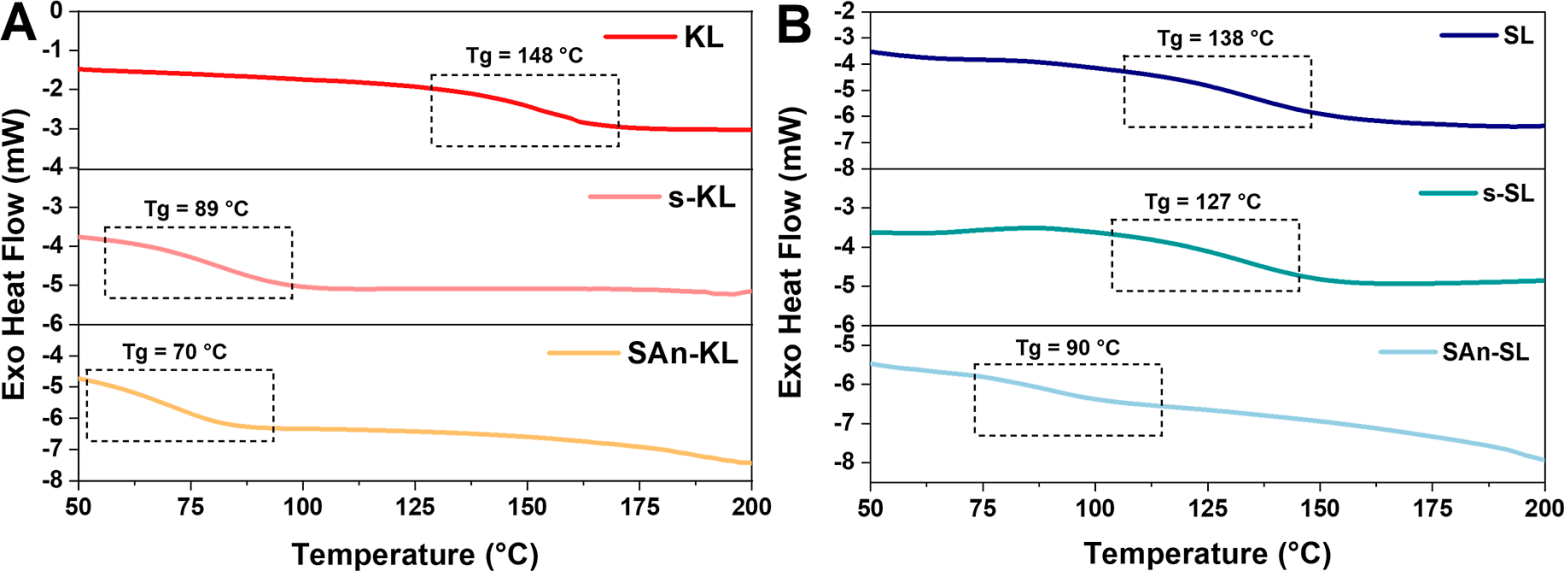
DSC traces of pristine, Soxhlet-extracted fractions and succinylated (A) kraft lignin and (B) soda lignin.

DSC measurements were carried out to determine the thermal transitions of the analyzed lignins. As shown in Figure 3, the *T*_g_ of the extracted soluble fractions for both kraft and soda lignin resulted significantly reduced by the extraction treatment. This can be attributed to different aspects. On the one side, the lower concentration of –OH groups found in s-KL and s-SL lignins with respect to KL and SL may lead to increased macromolecular mobility (viz., lower *T*_g_) as a result of the less pronounced intra-/intermolecular hydrogen-bonding interactions.^42^ On the other side, as confirmed by GPC analysis (Supporting Information, Table S1) and in line with previous reports,^40^ the extracted lignins are composed by high concentrations of low-molecular-weight fractions (preferentially extracted by the solvent), which are responsible for lowering the *T*_g_ of these systems via an internal plasticization effect. This phenomenon is even more evident when considering KL. Indeed, following the solvent extraction process, KL is subjected to a more marked drop of the glass transition temperature—more than 50 °C—compared to SL. This pronounced reduction can be ascribed to the significantly lower solubility in THF of KL with respect to SL (around 60^40^ and 85%,^42,43^ respectively). As a result of this different response, the extracted KL consists mainly of fractions with lower molecular weight and lower ability to establish intra-/intermolecular hydrogen bonds, consequently leading to a larger decrease of *T*_g_.^44^ The subsequent functionalization reaction with SAn led to a further slight reduction in *T*_g_ for both lignin fractions, most likely due to increased macromolecular mobility (free volume) resulting from the addition of the linear ester chain as a lateral substituent to the bulky lignin core.^33^

### 3.2 Study of the Curing Process

The fully oil-based reference PF resin, the biobased LPF resins based on pristine lignins KL and SL, and the biobased LPF resins containing succinylated SAn-KL and SAn-SL were prepared by first mixing the powdery phases (i.e., novolac and lignin in different weight ratios) and by adding in a second step the liquid phase (i.e., resole). From here on, the produced resins will be referred to as “LPF X *N*”, where X indicates the type of lignin used (KL, SL, SAn-KL, or SAn-SL) and *N* the weight percentage of lignin employed (5, 10, 20, 30, or 40) with respect to the total amount of novolac in the formulation (e.g., LPF SAn-KL 20 indicates the formulation in which 20 wt % of SAn-KL lignin is used).

Only formulations showing a gel content equal or greater than 95% after the curing step were considered for further characterization, as this represents the lower threshold for a thermoset material to be considered fully cross-linked.^45^ Gel content values (eq 3) are reported in Table 2 for each of the resins produced. The amount of cross-linked material (gel content) was found to slightly decrease by increasing the amount of lignin content, up to a certain lignin threshold, above which the gel value dropped below 95%. This threshold value determines the maximum amount of lignin that can be incorporated into the resin, which is higher for kraft lignin-based resins with respect to soda lignin-based resins. More importantly, the amount of successfully incorporated lignin in the cross-linked materials was found to increase with lignin functionalization, thus indicating higher reactivity of succinylated lignins versus their unmodified counterparts (more details will be discussed in the following sections). Interestingly, kraft lignin appeared to exhibit easier incorporation in the PF systems in comparison with soda lignin, in the case of both pristine and succinylated material. This behavior can be explained considering the higher amount of aromatic –OH functionalities found in the structure of KL and SAn-KL versus SL and SAn-SL, which are responsible to promote the reaction of free formaldehyde and HMTA with the free positions in the aromatic rings in lignin, leading to the condensed structure.^46^ In addition to this, S-type units (typical of grass lignins, such as SL) are less prone to react with the available cross-linkers (i.e., formaldehyde and HMTA) since the free C3 and C5 positions are linked to methoxy groups.^47^ The abundance of the S-units is confirmed by the presence of an absorption band located at around 1330 cm^–1^ in the FTIR spectra of soda lignins (Figure 2B), which is associated with the syringyl rings breathing.^48^ This signal is instead completely absent in the FTIR spectra of kraft lignins. Conversely, G-type units are prevalent in softwood kraft lignins,^49^ as demonstrated by the presence of two distinguishable signals related to C–H out-of-plane vibrations at 854 and 817 cm^–1^ in the FTIR spectra of kraft lignins (Figure 2A),^50^ which are more suitable for reaction with free formaldehyde and HMTA.^47^

**Table 2:** Gel Content, Temperature of the Exothermal Peak Associated with the Crosslinking Reaction (*T*_p_), Enthalpy of the Crosslinking Reaction (Δ*H*), Activation Energy (*E*_a_) According to Ozawa and Kissinger Model, and Gel Content of All the Resins Produced

sample	gel content (%)	*T*_p_ (°C)^a^	Δ*H* (J/g)^a^	*E*_a_ Ozawa (kJ/mol)	*E*_a_ Kissinger (kJ/mol)
PF	99	148	67	145	144
LPF KL 5	98	148	52	151	152
LPF KL 10	96	149	51	157	157
LPF KL 20	95	148	45	164	164
LPF KL 30	91				
LPF SAn-KL 5	97	147	66	147	148
LPF SAn-KL 10	96	149	63	164	165
LPF SAn-KL 20	96	147	48	165	166
LPF SAn-KL 30	95	148	39	174	175
LPF SAn-KL 40	88				
LPF SL 5	97	149	57	170	171
LPF SL 10	92				
LPF SAn-SL 5	97	149	62	152	149
LPF SAn-SL 10	96	150	61	192	195
LPF SAn-SL 20	89				

a Δ*H* and *T*_p_ reported refer to DSC performed at 5 °C/min.

Before the curing step, the behavior upon heating of the resinoid blends was studied by performing DSC analysis. In particular, DSC was used for both designing the optimal curing cycle and determining the activation energy of the resins according to the Ozawa and Kissinger models.

As evident from DSC analyses performed on uncured blends at the lower heating rate (i.e., 5 °C/min, Figure S3 in the Supporting Information), a main exothermic peak associated with the cross-linking reaction is always found at around 150 °C (see Table 2), irrespective of the lignin type and content in the system (endothermic contribution given by the evaporation of the condensation byproducts was found to be negligible). At this temperature, the excess of aldehydes together with the presence of unreacted methylol groups is responsible for the formation of methylene bridges, resulting in the formation of a condensed three-dimensional structure, in line with the literature.^51^ In parallel, as the temperature increases HMTA decomposes and, by reacting with phenol, it can form different intermediates (e.g., benzoxazine and benzylamine) able to break down and further react, yielding additional methylene linkages between the phenolic rings.^38^ In addition to the main exotherm, a secondary postcure exothermal peak is also observable at higher temperatures (∼200 °C), as a consequence of the presence of unreacted groups. Notably, a wide endothermic peak is also located at around 75 °C, which is attributable to the melting process of the novolac phase, as reported by the supplier of the commercial material. Considering these characteristics, the curing cycle of all resins was designed in three steps: the first one at 80 °C, to allow the precure novolac phase to adhere to the mold walls; the second one at 150 °C, to activate the formation of the methylene bridges; and the third one at 180 °C, to ensure the complete cross-linking of the thermoset material. The effectiveness of the thermal cycle applied (i.e., completed cross-linking reaction) could be confirmed based on the absence of residual exothermic peaks in DSC traces of cured materials (see Figure S7 in the Supporting Information) and by their excellent solvent resistance (i.e., high gel content values).

The enthalpy associated with the cross-linked reaction (Δ*H*) and the activation energy (*E*_a_) values calculated by applying the Ozawa and the Kissinger models are reported in Table 2, while their corresponding linear regression, for all the resins produced, are shown in the Supporting Information (Figures S4 and S5, respectively).

The Ozawa and Kissinger models are phenomenological kinetic models based on empirical data that can be obtained from multiple dynamic DSC scans. By assuming that the maximum rate of the cross-linking reaction occurs at the peak temperature (*T*_p_) of the exothermal signal recorded by DSC measurements^52^ (located at around 150 °C in this case), the activation energy can be derived applying eqs 1 or 2. To this end, DSC analyses were performed at different heating rates (i.e., 5, 10, and 20 °C/min) on the uncured resin blends.

As a general consideration, the two models appear to give comparable results, with curing activation energies in line with those obtained on commercial phenol-formaldehyde resins recently reported in the literature (144 kJ/mol).^38^ Interestingly, regardless of the lignin type used, lignin-substituted materials are characterized by a progressively higher *E*_a_ compared to that of the benchmark PF resin for increasing the lignin content. This result can be attributed to two different aspects. On the one side, the different ortho and/or para vacant sites in the aromatic rings of phenol versus lignin, which are responsible for reacting with formaldehyde or HMTA, yield different reaction mechanisms in the two systems.^49^ On the other side, the complex and bulky polyphenolic structure of lignin may be held accountable for the lower lignin reactivity compared to that of commercial novolac resins.^53^ The highest *E*_a_ values were found for soda lignin-based LPF resins, in line with previous discussions on the greater ability of kraft versus soda lignin to react in PF systems. In particular, kraft lignin-based materials showed *E*_a_ values between 147 kJ/mol (LPF SAn-KL 5) and 174 kJ/mol (LPF SAn-KL 30), as opposed to 152–192 kJ/mol in the case of soda lignin-based systems. The beneficial effect of lignin functionalization with SAn in the reaction for the formation of the phenol–formaldehyde network is more evident at lower lignin content (i.e., 5 wt %), where a significant decrease of the *E*_a_ can clearly be observed. This behavior may be ascribed to the higher amount of –COOH functionalities present in succinylated lignins, constituting active sites for the cross-linking reaction to occur through various possible condensation mechanisms (Figure 1) and acting as an accelerating moiety.^36^ Correspondingly, the enthalpy (Δ*H*) associated with the main cross-linking reaction (i.e., the one occurring at 150 °C) is lower in LPF resins than in commercial PF systems and it tends to decrease as the lignin content increases due to lower availability of reactive sites in lignins with respect to the common phenols.^12^ However, in agreement with the previous consideration about the acceleration effect of –COOH functionalities, Δ*H* values become higher when succinylated lignins are used in the LPF resin formulations (both in the case of soda and kraft).

The curing process of PF and LPF resins was also studied by means of FTIR spectroscopy (FTIR spectra registered for the reference PF thermoset material and representative spectra of all LPF resins containing 5 wt % of lignin, before and after the curing process, are available in the Supporting Information, Figure S6), with no substantial differences detectable in the IR spectra of lignin-based versus unmodified PF resins, in line with previous literature (discussion on FTIR spectra is reported in the Supporting Information as well).^12,54^ This evidence constitutes further proof of the effectiveness of the cross-linking step and, at the same time, the suitability of lignin as a biobased reagent in PF systems.

### 3.3 Thermal and Thermo-Oxidative Characterization of the Cured Resin Systems

Fully cured resins based on lignin (gel content ≥ 95%, see Table 2) were characterized by means of DSC and TGA measurements in order to compare their thermal and thermo-oxidative behavior in reference to the base PF resin.

**Figure 4:**
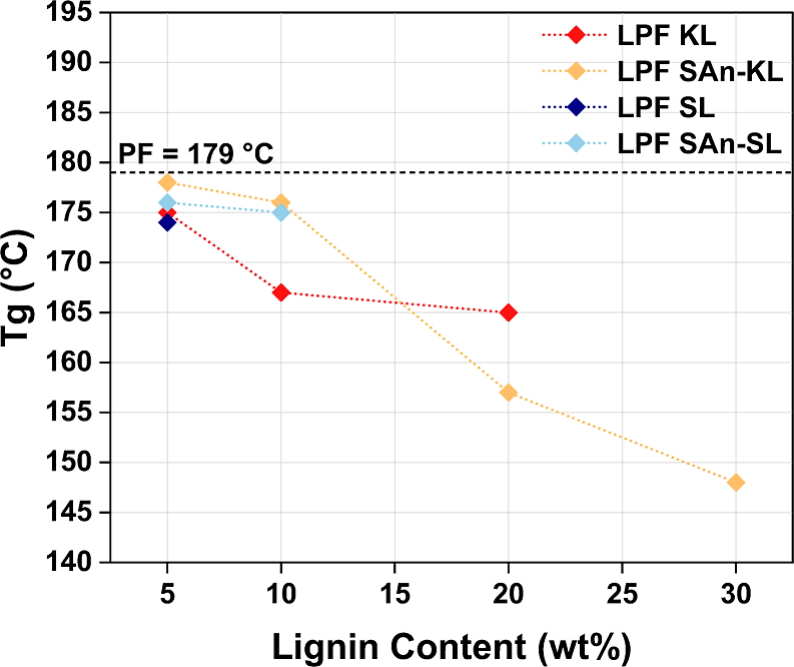
*T*_g_ values of LPF resins as a function of lignin wt % in the novolac phase.

DSC analyses were carried out to determine the *T*_g_ of the cured resins. The resulting values are reported in Figure 4 as a function of lignin content (full DSC traces can be found in Supporting Information, Figure S7). For each of the analyzed resins, a single *T*_g_ was always detected, thus excluding phase segregations in the cured materials. The benchmark PF resin was found to exhibit a *T*_g_ value of ∼180 °C in accordance with literature.^55^ LPF resins incorporating 5 wt % lignin were all characterized by comparable *T*_g_ values (close to that of the reference PF resin), slightly higher in the presence of SAn lignins (i.e., 178 and 176 °C for LPF SAn-KL 5 and LPF SAn-SL 5, respectively). Up to 10 wt % lignin, the effect of succinylation appeared beneficial in maintaining a higher *T*_g_ value (∼175 °C) with respect to LPF formulations based on pristine lignin. This behavior may be associated with the enhanced interactions of the SAn lignin within the PF system, resulting in a more compact macromolecular network characterized by lower degrees of freedom (viz., increased *T*_g_) and expected higher cross-linking degree.^56^ On the contrary, for higher lignin content (i.e., 20 and 30 wt %), the *T*_g_ was found to decrease by 15 and 25 °C, respectively, thus highlighting the plasticization effect provided by the large excess of SAn-KL, the latter exhibiting a *T*_g_ of 70 °C (see Section 3.1).

**Figure 5:**
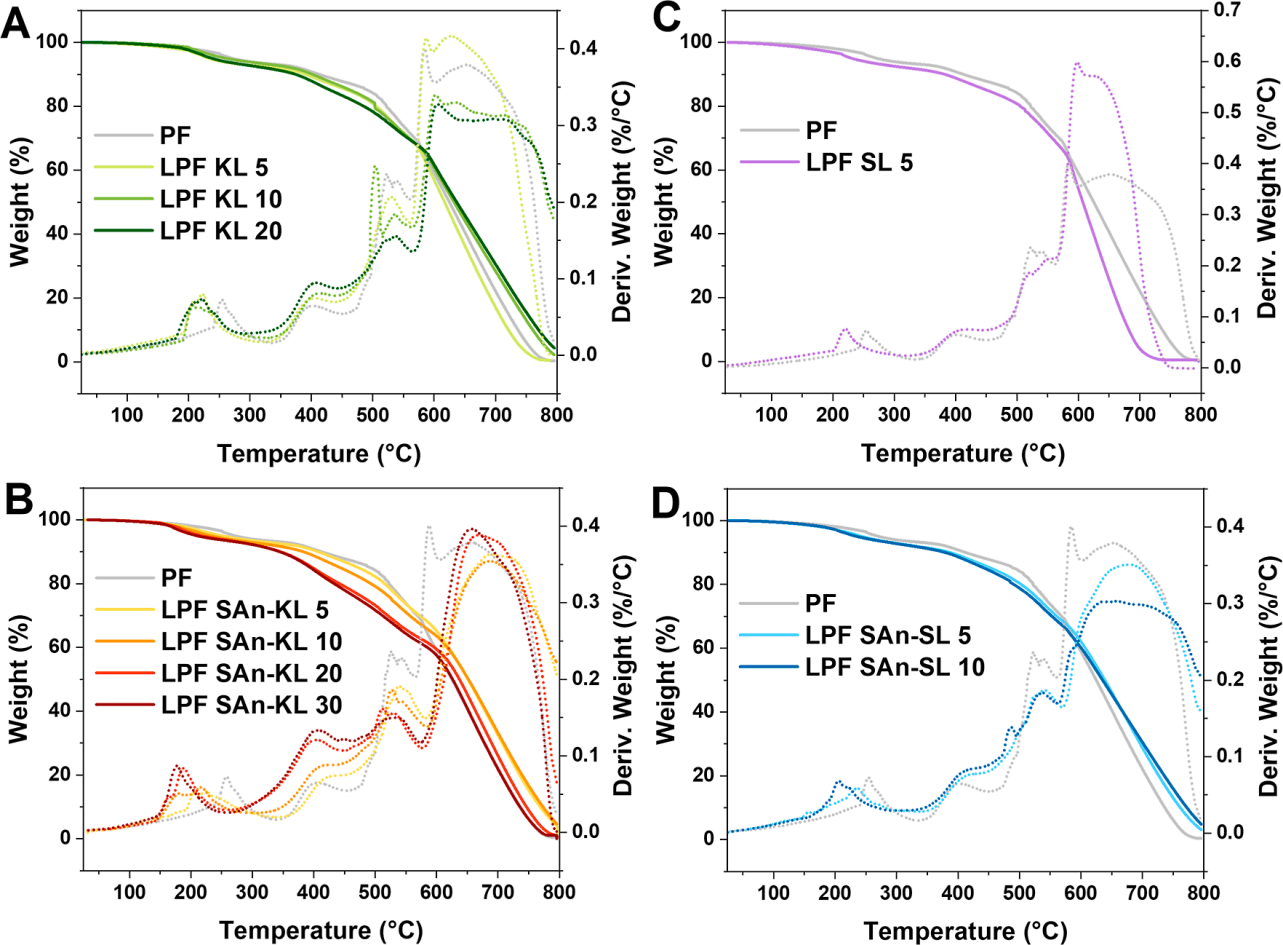
Thermogravimetric traces (solid lines) and DTG curves (dashed lines) in an air atmosphere of (A) pristine kraft lignin-based resins LPF KL, (B) succinylated kraft lignin-based resins LPF SAn-KL, (C) pristine soda lignin-based resins LPF SL, and (D) succinylated soda lignin-based resins LPF SAn-SL. PF commercial resin is also shown as reference.

To assess the thermo-oxidative stability and to study the degradation behavior of the different resin systems, TGA measurements were performed in an air atmosphere. The thermogravimetric mass loss traces (TG) as a function of temperature for all LPF materials, together with the reference PF resin, are reported in Figure 5, where the thermogravimetric derivative (DTG) curves are also shown (dashed lines). As can be observed from TG curves and DTG peaks, all of the resins were found to undergo similar multistep degradation events. The first significant mass loss, known as *postcure*, could be observed at temperatures below 300 °C, where the removal of small terminal groups (e.g., aldehyde carbonyl groups) and the evaporation of the low molecular weight condensation byproducts typically occurs.^57^ In this temperature range, a slightly higher mass loss (i.e., 5%) was observed in lignin-based systems than in the commercial reference PF, likely due to the higher abundance of volatile species (physically adsorbed water) in LPF resins, as a result of the highly hygroscopic nature of lignin.^58^ In addition to this, in the 150–300 °C temperature range lignin undergoes breaking of α- and β-aryl-alkyl-ether linkages, as well as decarboxylation reactions, which can also be responsible for a slightly lower thermal stability.^33^

At higher temperatures (300–600 °C), the so-called *thermal reforming* degradation process takes place. This phenomenon involves the breakdown of ether bonds formed upon cross-linking reaction in the resin into more stable methylene bridges.^57,59^ Also in this phase, the material loss by LPF resins was found to be slightly more pronounced than in PF, due to the possible rupture of intramolecular carbon–carbon linkages within the lignin macromolecular structure.^33^

In the last step (*ring stripping*, from 600 to 800 °C), the mass loss occurring to the materials can be attributed to the release of carbon monoxide and methane formed upon degradation of methylene bridges.^12,59^ This thermal event constitutes the most significant degradation phenomenon occurring for all cured materials investigated in this work, as evident from the DTG peak associated with the maximum mass loss rate in this temperature range. Very interestingly, in the case of LPF materials, this peak (and the corresponding TG curve) is shifted to higher temperatures, clearly indicating the higher thermal stability of the lignin-based network versus the reference PF material.

**Figure 6:**
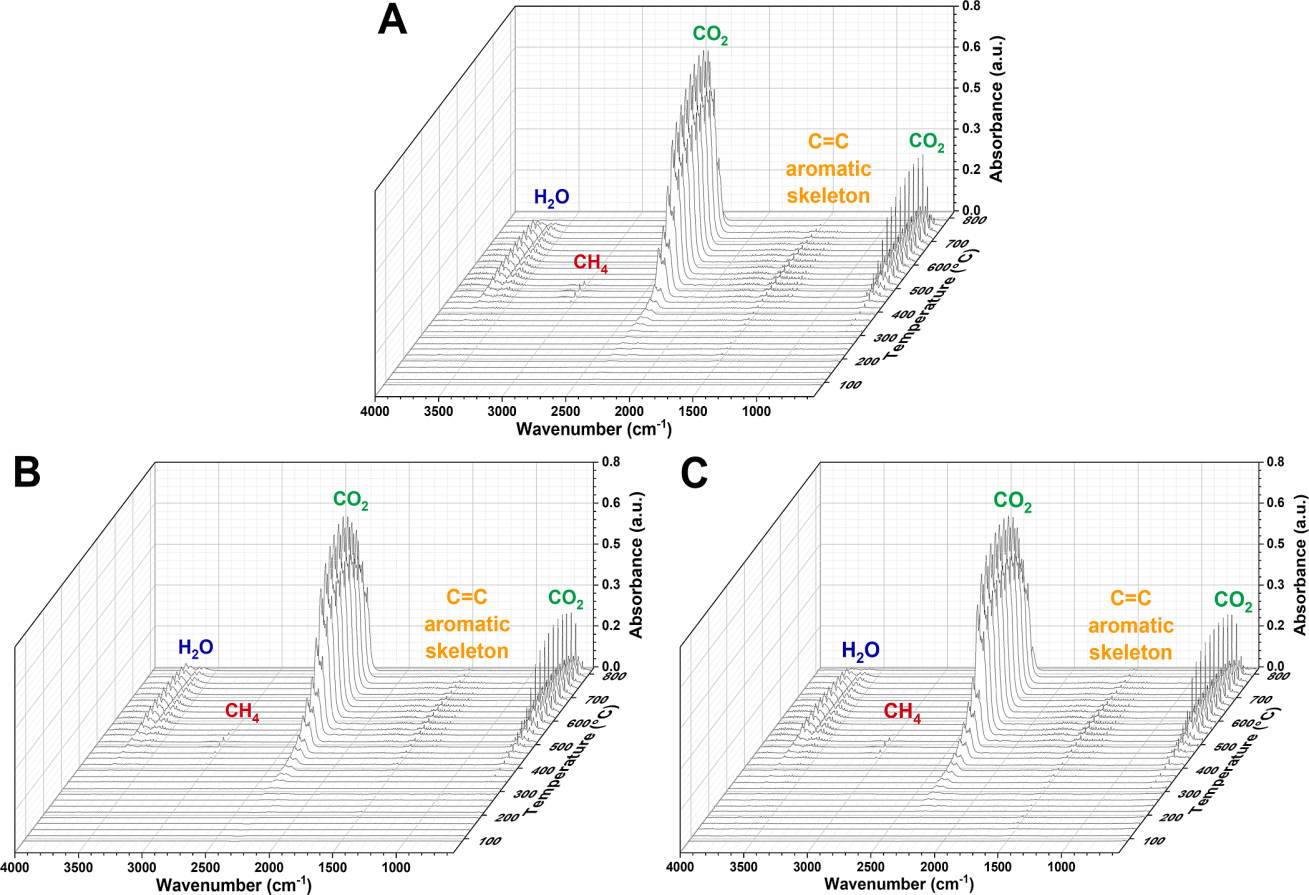
FTIR stack plots of gaseous products evolved from (A) PF, (B) LPF SAn-KL 10, and (C) LPF SAn-SL 10 as a function of temperature from TGA-FTIR coupled measurements.

In order to better understand the thermo-oxidative degradation process occurring in the resins, FTIR analyses were performed on the gases evolving from the cross-linked materials during TGA measurements by means of a TGA-FTIR coupled system. The stack FTIR spectra recorded for representative PF, LPF SAn-KL 10, and LPF SAn-SL 10 systems are reported in Figure 6. In line with TGA measurements (Figure 5), both PF and LPF resins appear to follow a similar thermo-degradative trend in which the main mass loss events are encountered between 400 and 700 °C.

More specifically, the FTIR absorption band found in the 4000–3200 cm^–1^ region (stretching vibration of the O–H bonds) can be associated with water release during temperature increase. Since this band is only detectable at a high temperature (i.e., >300 °C), the release of water in this case may be attributed to two different mechanisms: phenol OH–OH condensation to yield diphenyl ether linkages in the 300–400 °C range; further reaction occurring at 500–600 °C between phenolic OH and methylene bridges.^60^ The peak visible at around 3050 cm^–1^, which appears in the 400–600 °C temperature range, is instead ascribable to stretching vibrations in C–H bonds, likely resulting from the presence of CH_4_ generated by the decomposition of methoxy groups.^61^ CO_2_ evolution is denoted by the broad peaks found between 2500 and 2000 and 800–650 cm^–1^ as a result of breakage of ether bridges, dissociation of diaryl ether bonds^61^ or, at lower temperatures, decomposition of carboxylic acids present in the resin.^62^ The intensity of these signals increases with temperature, suggesting a more intense degradation phenomenon accompanied by a marked release of volatile species. Finally, the weak absorption band detected in the region around 1510 cm^–1^ can be attributed to C=C stretching vibrations of the aromatic skeleton, suggesting that low-molecular-weight phenolic compounds are released at temperatures higher than 400 °C.^62^

### 3.4 Mechanical Behavior of the Cured Resin Systems

To better understand the physical properties of lignin-derived resins in view of their potential application (e.g., as binder for composites and for abrasive tools, as wood adhesive), a comprehensive mechanical characterization of the thermoset materials produced was performed through DMA and microindentation tests.

**Figure 7:**
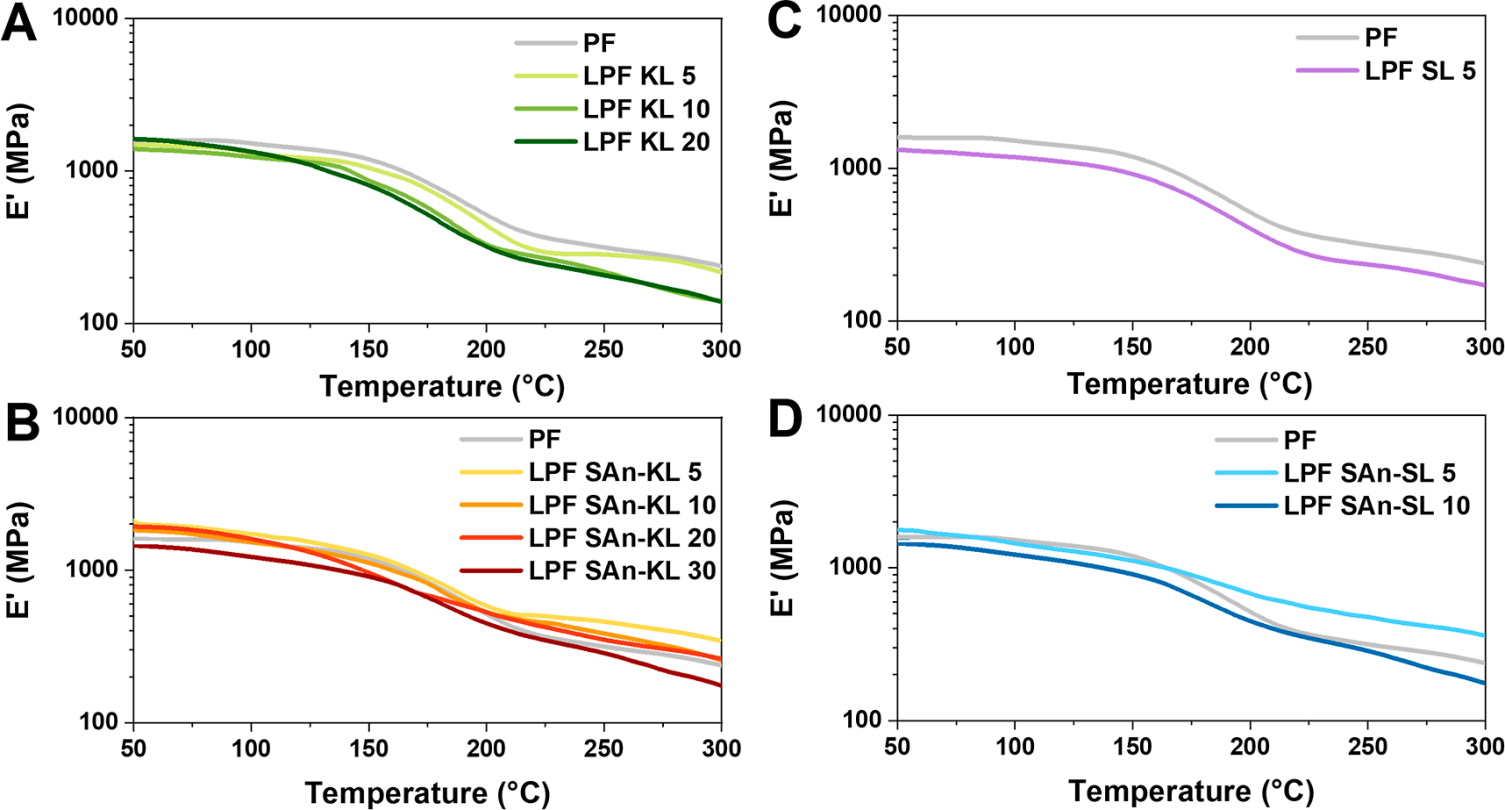
Storage modulus *E*′ vs temperature as obtained from DMA measurements on (A) pristine kraft lignin-based resins LPF KL, (B) succinylated kraft lignin-based resins LPF SAn-KL, (C) pristine soda lignin-based resins LPF SL, and (D) succinylated soda lignin-based resins LPF SAn-SL. The commercial resin (PF) is also shown as reference.

DMA measurements were carried out via dynamic temperature scans in the tensile mode to study the effect of lignin functionalization with SAn on the thermomechanical response of the cured resins, at varying lignin-substitution content. The storage modulus *E*′ as a function of temperature for the different lignin-based systems in comparison with the reference PF material is presented in Figure 7, while the values of storage modulus *E*′ at room temperature (*E*_RT_′) and cross-linking density obtained according to eq 5 are collected in Table 3.

**Table 3:** Mechanical Properties of PF and Lignin-Based Resins^a^

sample	*E*_RT_′ (GPa)	*Ν* (10^–^^3^ × mol/cm^3^)	*H*_IT_ (N/mm^2^)	η_IT_ (%)	*C*_IT,1_ (%)	*C*_IT,2_ (%)
PF	1.62	28.3	741 ± 140	71.0 ± 3.6	1.4 ± 0.1	(−) 18.1 ± 3.4
LPF KL 5	1.56	22.8	729 ± 167	67.5 ± 6.9	1.6 ± 0.5	(−) 18.5 ± 3.5
LPF KL 10	1.41	22.9	732 ± 100	74.8 ± 5.0	1.2 ± 0.2	(−) 23.1 ± 2.1
LPF KL 20	1.64	21.4	703 ± 107	66.9 ± 3.0	1.7 ± 0.5	(−) 16.1 ± 1.7
LPF SAn-KL 5	2.06	44.2	712 ± 109	74.8 ± 6.3	1.9 ± 0.5	(−) 19.2 ± 3.0
LPF SAn-KL 10	1.83	40.9	798 ± 129	76.0 ± 4.0	1.6 ± 0.4	(−) 22.3 ± 2.9
LPF SAn-KL 20	1.90	36.1	800 ± 100	70.8 ± 2.9	1.6 ± 0.5	(−) 25.7 ± 2.2
LPF SAn-KL 30	1.84	32.3	779 ± 160	73.9 ± 3.0	1.9 ± 0.4	(−) 26.2 ± 1.9
LPF SL 5	1.32	21.1	754 ± 114	67.5 ± 7.0	1.3 ± 0.4	(−) 15.6 ± 3.4
LPF SAn-SL 5	1.81	39.5	689 ± 103	69.5 ± 1.7	1.6 ± 0.4	(−) 16.7 ± 1.6
LPF SAn-SL 10	1.84	36.3	797 ± 120	76.9 ± 4.9	1.5 ± 0.3	(−) 18.5 ± 2.5

a Storage modulus at room temperature as obtained through DMA measurements in tensile mode (*E*_RT_′), crosslinking density (ν), Vickers hardness (*H*_IT_), elastic portion of indentation work (η_IT_), indentation creep (*C*_IT,1_), and recovery creep (*C*_IT,2_). Average values of six measurements are reported, together with the corresponding standard deviation.

LPF resins are characterized by a room-temperature modulus comparable to that of the reference PF material (∼1.5–2 GPa), in line with analogous phenolic resin systems recently reported in the literature.^63^ The incorporation of SAn-lignin in the resin formulation appears to yield slightly higher *E*_RT_′, suggesting a more effective incorporation of succinylated lignin in the PF system, in line with previous considerations. The same beneficial effect of lignin functionalization is also found on the cross-linking density of the cured resins. While slightly lower values of ν are found for LPF systems incorporating unmodified lignin (∼21–23 × 10^–3^ mol/cm^3^ for all LPF KL and LPF SL systems) with respect to the benchmark commercial material (ν = 28.3 × 10^–3^ mol/cm^3^), opposite trends are found for SAn-lignin-based formulations. Indeed, all LPF-SAn systems exhibit ν values higher than those found for PF, with a maximum in the case of LPF SAn-KL 5 (ν = 44.2 × 10^–3^ mol/cm^3^). This result represents clear evidence of the active role played by SAn-lignins in the formation of the cross-linked network, likely due to possible additional condensation reactions enabled by the more reactive –COOH groups incorporated upon functionalization with SAn. Indeed, the presence of ester groups in the PF system could lead to the formation of additional cross-linking bonds in the form of diester bridges. Additionally, the ester may also react with normally unreactive meta sites of phenol rings, promoting the formation of new bonds in the resin.^34,36,64^ These findings can also explain the higher gel content registered for esterified lignin resins (Section 3.2).

Microindentation tests were also performed on PF and LPF resins to evaluate their surface hardness and their viscoelastic behavior (see Figures S9 and S10 in the Supporting Information for representative microindentation curves). The average Vickers hardness (*H*_IT_), elastic portion of indentation work (η_IT_), indentation creep (*C*_IT,1_), and recovery creep (*C*_IT,2_) of the reference PF resin and LPF resins are also reported in Table 3. The surface behavior of a thermoset is highly influenced by its degree of cross-linking and its *T*_g_. Indeed, high hardness values are typically found for polymeric materials showing a high cross-linking degree and high *T*_g_.^65^ Data collected through microindentation tests showed that the Vickers indentation hardness *H*_IT_ for all LPF samples is comparable (in some cases slightly higher) to that of the benchmark PF resin, being the latter ∼740 N/mm^2^. These results represent further evidence of the high cross-linking degree reached in the lignin-substituted samples. Interestingly, despite the lower *T*_g_ values recorded for LPF samples substituted with 20 and 30 wt % of SAn-KL, their surface hardness remains in line with that of the pristine PF resin, demonstrating the optimal incorporation of high concentrations of lignin in the PF systems. Finally, in accordance with the high stiffness and *T*_g_ characterizing these thermosetting materials, a large fraction of the indentation work was found to be of elastic nature. Indeed, all cured resin systems exhibited η_IT_ values in the 67–77% range, confirming the high extent of curing of these materials, which enables efficient recovery of small deformations and prospects excellent creep recovery after load during operation. Indeed, low creep values during the indentation process were always recorded (*C*_IT,1_ in the 1–2% range), while higher recovery values were obtained after load release (*C*_IT,2_ in the 16–26% range). These trends did not appear to be affected by the type and amount of lignin used, further proving its successful incorporation in the different formulations (as also evidenced by cryo-fracture SEM images on all systems; see Figure S11 in the Supporting Information).

The mechanical response of the lignin-derived resins was also tested from an applicative point of view by performing wood-joint adhesion tests on three-ply specimens according to BS ISO 6237:2003 (see the Supporting Information, Figure S8). For all the adhesives tested, 100% wood failure of the plywood occurred—at a stress higher than 15 MPa—with no detectable exposed adhesive. This result denotes the excellent performance as wood adhesives of all the tested LPF resins, whose adhesion strength resulted at least on par with that of commercial formulations, and in line (or higher) than what recently reported in the literature.^12,66^

Based on the experimental evidence discussed above, kraft lignin proved to be easier to incorporate into the phenolic resin system as compared with soda lignin. Indeed, a higher gel content, lower activation energy of the cross-linking reaction, and higher cross-linking density were obtained for materials based on kraft lignin. Lignin functionalization with SAn was shown to allow the incorporation of a larger amount of lignin in the formulations, to yield an increased cross-linking density, and to improve the thermal stability of the macromolecular network. With a view to potential future applications, the choice of lignin type should be made according to the target end use. Taking as a reference example the field of abrasive tools, phenolic resins typically used as binders are preferably characterized by a high cross-linking density, as well as by excellent thermo-oxidative stability. In this specific case, the optimal choice would be SAn-functionalized lignins. Conversely, considering a different target sector such as that of phenolic adhesives, a lower degree of cross-linking could enable for a larger flexibility of the adhesive layer and greater resistance to adhesive failure. In addition, being that the working temperature is typically around room temperature in these cases, thermal and thermo-oxidative stability play a less central role, so that the use of pristine lignins could also be considered as a viable option.

## 4 Conclusions

Lignin-based phenolic resins were developed and are presented in this study. Kraft and soda lignin were esterified by means of succinic anhydride and used as a partial replacement of the novolac component in commercial PF formulations in different amounts. FTIR and ^31^P NMR analyses revealed the increase in concentration of carboxyl groups in lignin upon functionalization (from 0.4 to 2.9 mmol/g and from 0.8 to 2.4 mmol/g in kraft and soda lignin, respectively), as a result of the successful reaction with succinic anhydride. The activation energy of the curing reaction for the thermosetting formulations was found to increase slightly with increasing lignin content. However, lower values were observed in the case of succinylated-lignin-based resins, pointing out the acceleration effect on the cross-linking reaction provided by esterified lignin. In line with this evidence, the enthalpy associated with the main cross-linking reaction was shown to decrease for higher lignin contents but was also found to increase when the SAn-functionalized lignin was employed in the PF formulations. After the curing process, gel content measurements showed that succinylated lignin is more reactive compared to pristine lignin and that kraft lignin provides an easier and more efficient incorporation into the cured systems. The highest degree of lignin substitution in the PF system was found with SAn-KL, which showed a gel content value > 95% also at 30 wt % substitution degree. The thermal response of LPF resins highlighted a comparable behavior to commercial phenolic resins, with the presence of a single *T*_g_ in the 150–180 °C temperature range, and excellent thermo-oxidative stability at high temperatures (typically, above 600 °C), irrespective of lignin concentration. Interestingly, lignin functionalization with SAn allowed to achieve higher values of cross-linking density versus reference commercial phenolic systems, likely due to the formation of additional permanent intermolecular bonds such as diester bridges. The high extent of cross-linking in all lignin-based systems was further confirmed by microindentation measurements, which highlighted excellent response in terms of surface hardness, indentation modulus, and elastic response.

This work provides the first demonstration of the incorporation of SAn-functionalized lignin in the formulation of high-performance phenolic resins and paves the path for the definition of design guidelines for the development of future high-performance, biobased thermosets for application in the context of advanced and sustainable manufacturing.

## Supporting Information Available

SEM images of lignins; ^31^P NMR spectra of pristine, THF-soluble, and succinylated lignins; GPC analysis of lignin samples; DSC traces of uncured and cured resins; Ozawa and Kissinger linear regression of resins; FTIR of uncured and cured resins; characteristic mass loss temperature from TGA; geometry used for wood joint adhesion tests; example of typical microindentation cycle; representative example of microindentation cycle on resins; and SEM images of resins (PDF)
