# Supplementary material for: Elucidating the Role of Lignin Type and Functionality in the Development of High-Performance Biobased Phenolic Thermoset Resins
Source: ACS Appl Polym Mater. 2024 Jan 10;6(2):1191–203. doi: 10.1021/acsapm.3c02136 (PMC10825815; doi:10.1021/acsapm.3c02136)
Supplement: Supplementary file 1 — ap3c02136_si_001.pdf [file ap3c02136_si_001.pdf]

Supporting Information

Elucidating the role of lignin type and functionality in the development of high-performance bio-based phenolic thermoset resins

Emanuela Bellinetto[a], Nicholas Fumagalli[a], Matilde Astorri[a], Stefano Turri[a], Gianmarco Griffini[a]*

[a]Department of Chemistry, Materials and Chemical Engineering "Giulio Natta", Politecnico di Milano, Piazza Leonoardo da Vinci 32, 20133 Milano, Italy

*Corresponding author: gianmarco.griffini@polimi.it

Table of contents

Characterization of lignins....S3

Study of the curing process of resins....S5

Thermal and thermo-oxidative characterization of resins....S10

Mechanical behaviour of resins....S12

Microstructure of cured resins....S14

References....S16

Index of Figures

Figure S1. SEM images of lignins....S3

Figure S2. ^{31}P-NMR spectra of pristine, THF-soluble and succinylated lignins....S4

Figure S3. DSC traces of uncured resins....S5

Figure S4. Ozawa linear regression of resins....S6

Figure S5. Kissinger linear regression of resins....S7

Figure S6. FTIR of uncured and cured resins....S8

Figure S7. DSC traces of cured resins....S10

Figure S8. Geometry used for wood joint adhesion test....S11

Figure S9. Example of typical microindentation cycle....S12

Figure S10. Representative example of microindentation cycle on resins....S13

Figure S11. SEM images of PF and of some representative LPF resins....S14

Index of Tables

Table S1. GPC analysis of lignin samples....S4

Table S2. Characteristic temperatures of TGA....S10

Characterization of lignins

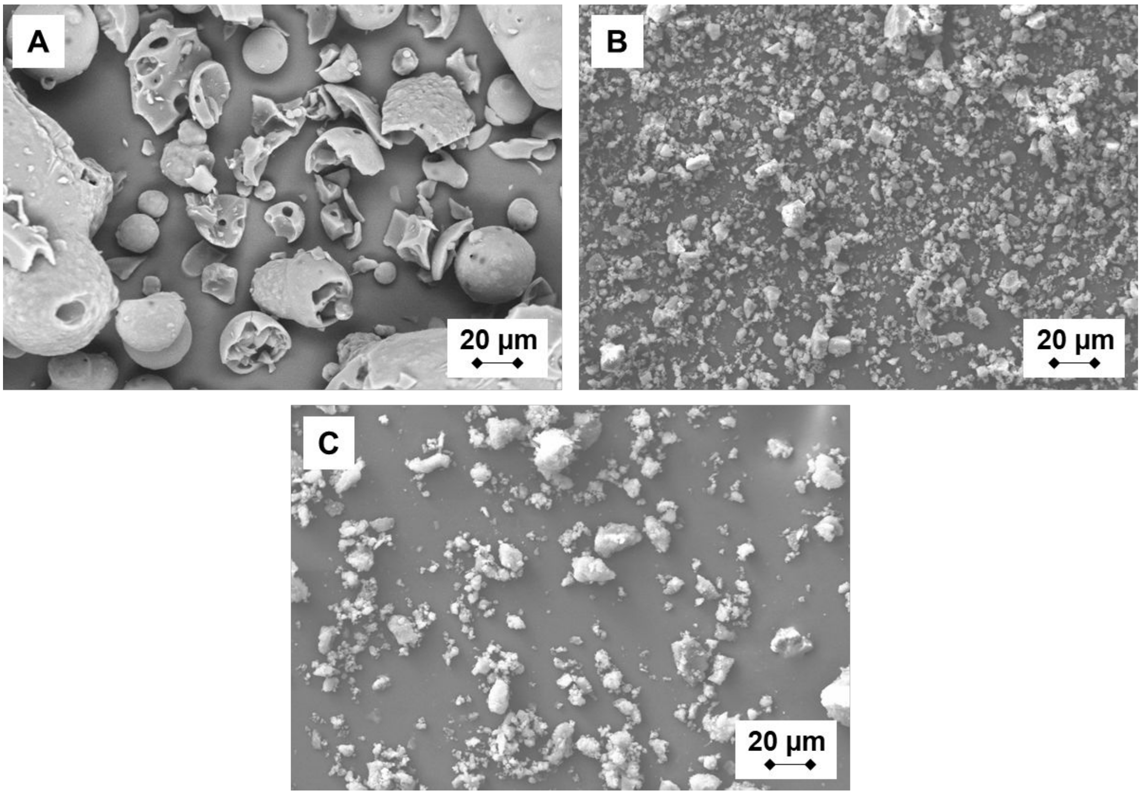

Figure S1 SEM images of **(A)** pristine KL **(B)** milled KL and **(C)** pristine SL.

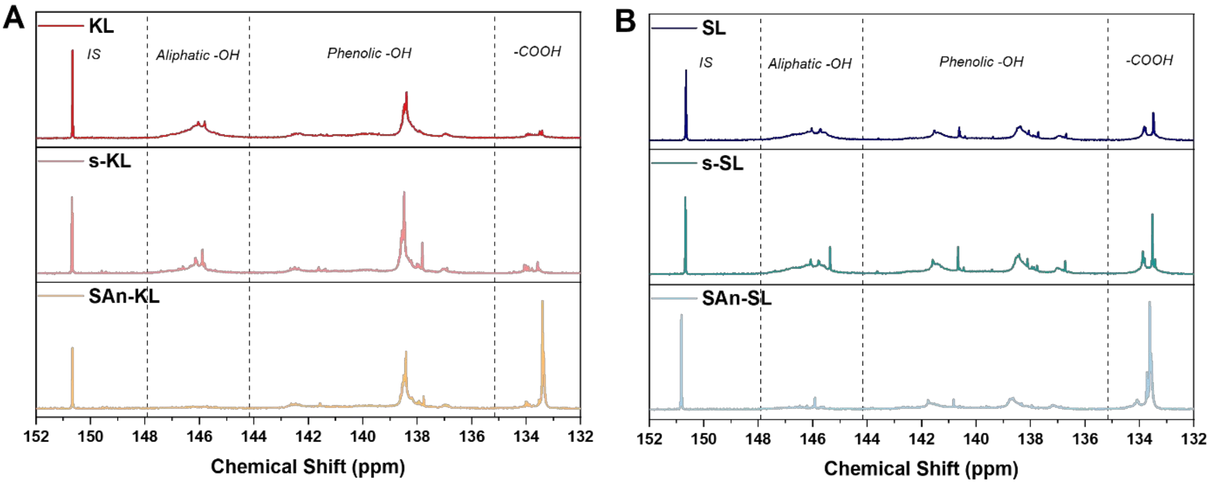

Figure S2 ^{31}P-NMR spectra of **(A)** pristine kraft lignin (KL), THF-soluble kraft lignin (s-KL), succinylated kraft lignin (SAn-KL) and **(B)** soda lignin (SL), THF-soluble soda lignin fraction (s-SL) and succinylated soda lignin (SAn-SL). Integration intervals are shown for reference. IS indicates the peak associated with the internal standard.

Table S1 GPC analysis of all lignin samples.

Sample	Mn (g/mol)	Mw (g/mol)	PDI (g/mol)
KL	1840	6840	3.7
s-KL	975	1570	1.7
SAn-KL	1003	1273	1.3
SL	1550	8320	5.4
s-SL	820	1260	1.5
SAn-SL	918	1229	1.3

A Waters 510 HPLC system equipped with a refractive index detector was used for gel permeation chromatography (GPC) analysis, to determine the molecular weight and molecular weight distribution of the lignins before and after fractionation and functionalization. THF was used as eluent. The analyzed lignin samples (volume 200 µL, concentration 1 mg/mL in THF) were injected into a system of three columns connected in series (Ultrastyragel HR, Waters–dimensions 7.8 mm × 300 mm) and the analysis was performed at 30 °C at a flow rate of 0.5 mL/min. The GPC system was calibrated against polystyrene standards in the 102–104 g/mol molecular weight range. To allow complete solubility in the THF eluent, before the analysis the parent lignins KL and SL were acetylated.[S1] The estimation of the number-average and weight-average molecular weights (Mn and Mw, respectively) of the obtained lignin fractions was performed excluding the signals related to the solvent (THF) and the solvent stabilizer (butylated hydroxytoluene), visible at long elution times (>29.5 min).

Study of the curing process of the resins

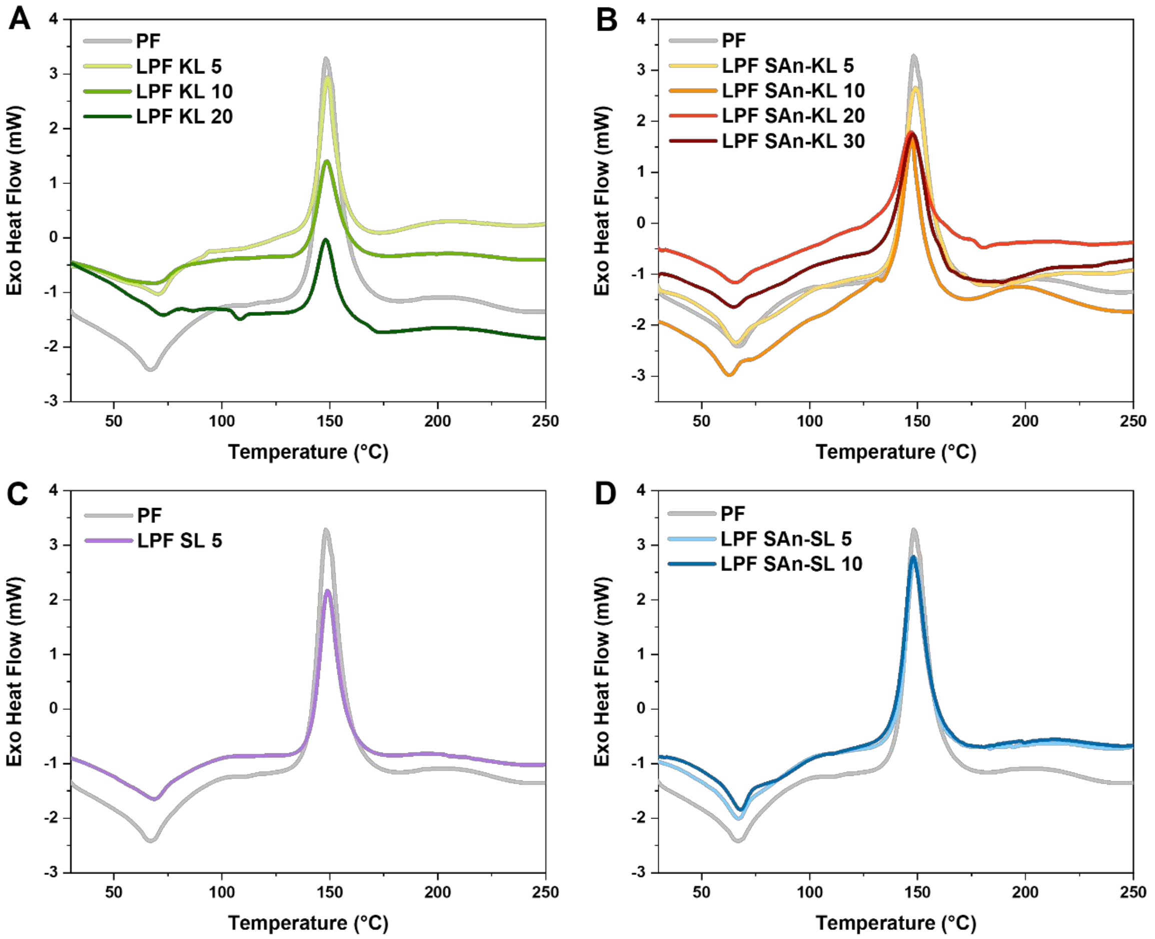

Figure S3 DSC at 5 °C/min of **(A)** pristine kraft lignin-based resins, **(B)** succinylated kraft lignin-based resins, **(C)** pristine soda lignin-based resins and **(D)** succinylated soda lignin-based resins.

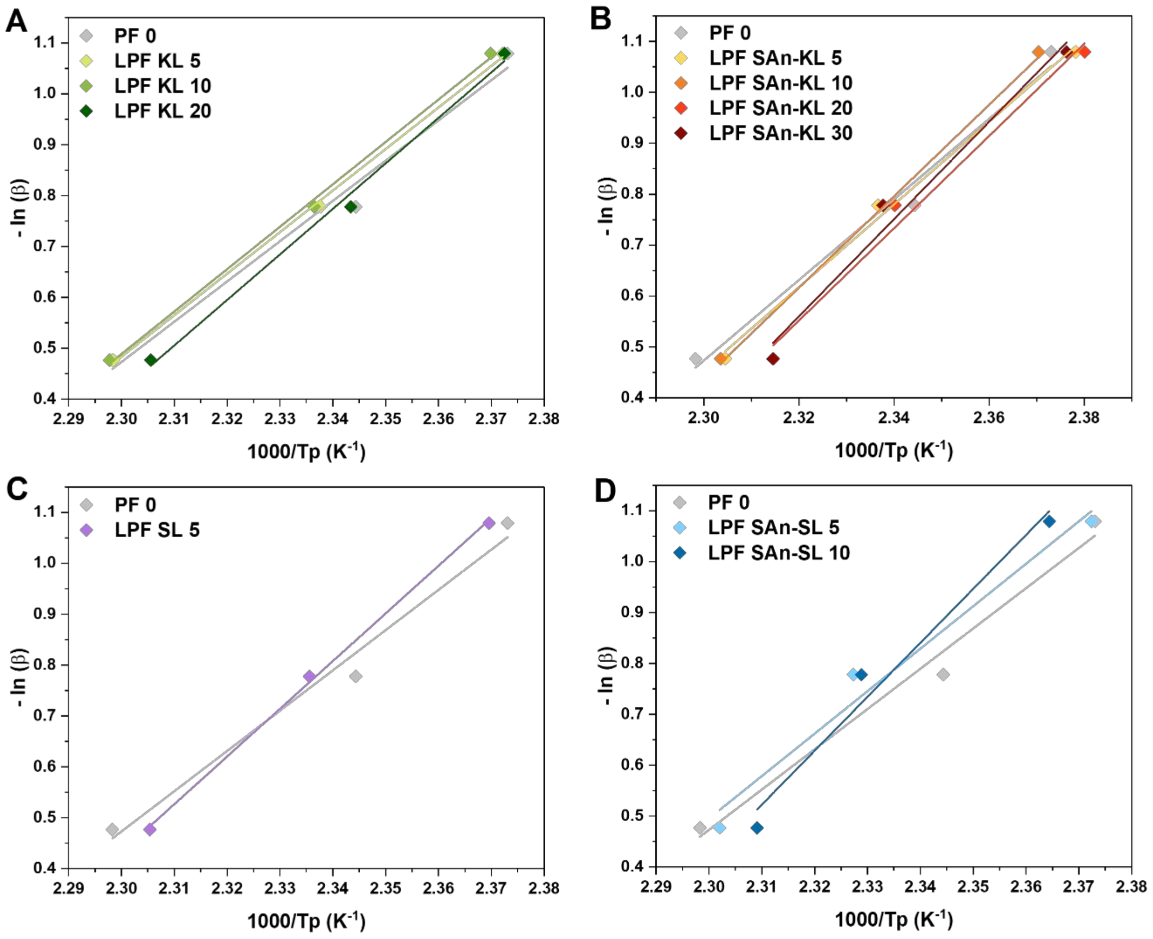

Figure S4 Ozawa linear regression for the evaluation of the activation energy associated with the crosslinking reaction of (A) pristine kraft lignin-based resins, (B) succinylated kraft lignin-based resins, (C) pristine soda lignin-based resins and (D) succinylated soda lignin-based resins.

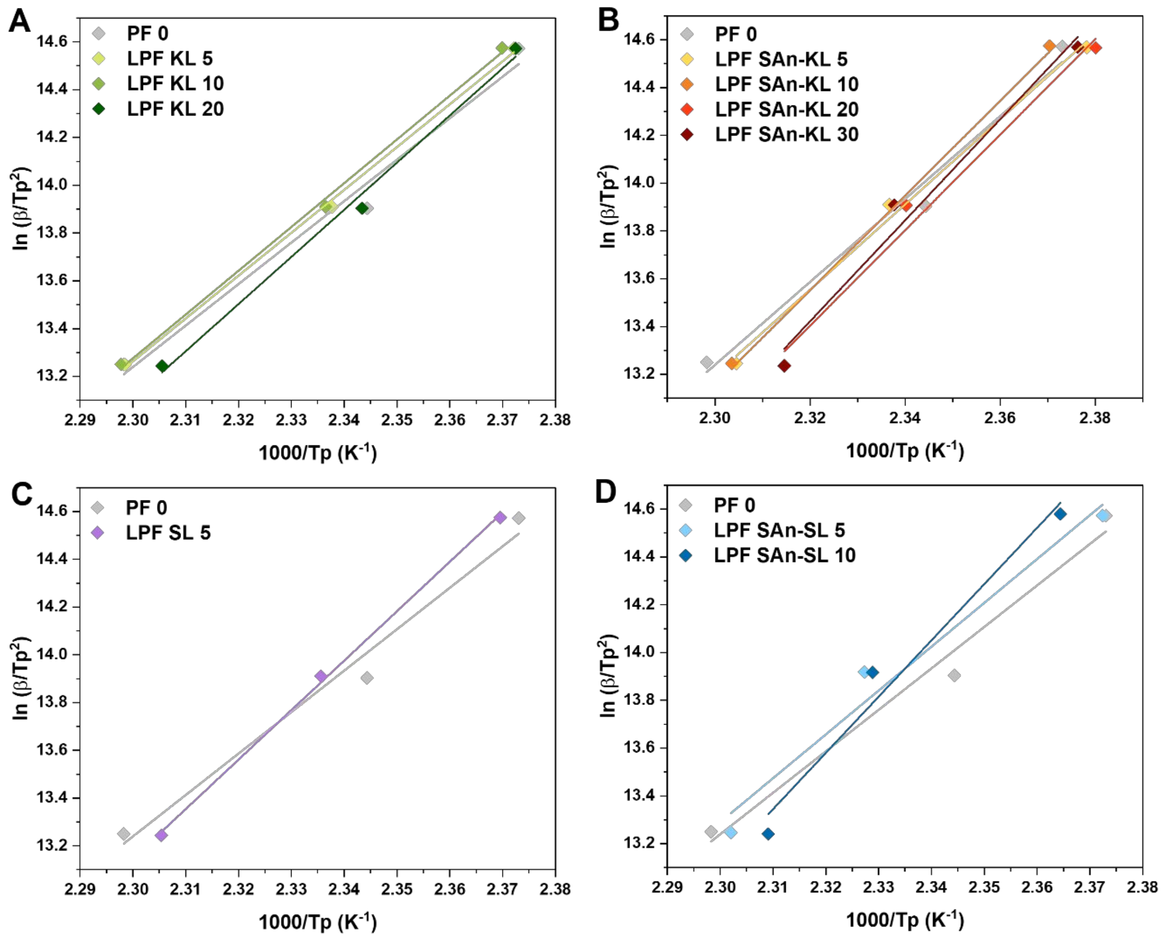

Figure S5 Kissinger linear regression for the evaluation of the activation energy associated with the crosslinking reaction of (A) pristine kraft lignin-based resins, (B) succinylated kraft lignin-based resins, (C) pristine soda lignin-based resins and (D) succinylated soda lignin-based resins.

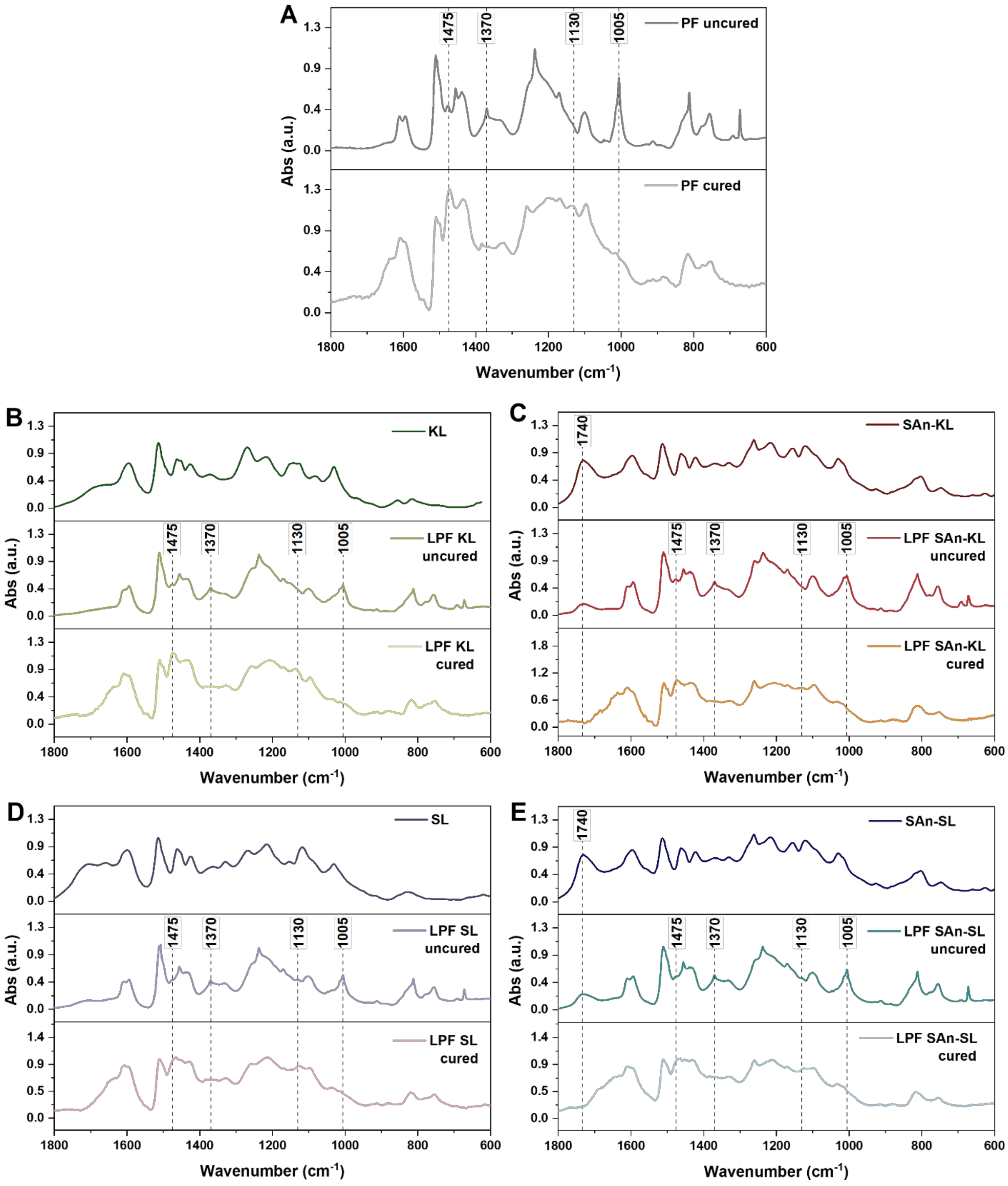

Figure S6 FTIR of uncured resinoid blends and cured resins in the region 1800-600 cm^{-1} of **(A)** reference resin PF, **(B)** LPF based on pristine kraft lignin, **(C)** LPF based on succinylated kraft lignin, **(D)** LPF based on pristine soda lignin and **(E)** LPF based on succinylated soda lignin.

Comparing all the spectra before and after the application of the curing cycle, some common changes in the signals recorded can be found. The most evident effect of curing process is the disappearance of the peak located around 1000 cm^{-1}, typically attributed to the stretching vibration of C-O bond in methylol group.[S2,S3] As curing proceeds, in fact, those species are involved in the formation of methylene bridges. This is confirmed by the appearance of a new band around 1475 cm^{-1}, correlated to the deformation of the formed CH_2 groups.[S4,S5] At the same time, the reduction of the signal located at 1370 cm^{-1} is observed, assigned to aromatic O-H in plane bending, as a consequence of further condensation reactions.[S4] Additionally, in all the cured resins, a new peak at 1130 cm^{-1} appears. This latter is attributed to the formation of a different type of crosslinking linkages (i.e., dimethyl-ether bridges CH_2-O-CH_2), as a result of the polycondensation reaction between hydroxymethyl groups and phenolic rings.[S5] In the case of SAn-functionalized lignins, the peak located at 1740 cm^{-1}, associated with C=O stretching of carbonyl bond and clearly visible in the spectra of uncured LPF SAn-KL and LPF SAn-SL, drastically decreases after crosslinking. This could likely be related to the decarboxylation of carbonyl bond, induced by the temperature/pressure conditions reached during the thermal cycle.[S6] Lastly, other characteristic peaks of all cured resins spectra are found at 820 and 760 cm^{-1}, owing to the out-of-plane bending vibrations of C-H in aromatic rings when *ortho–ortho* and *ortho–para* connections are established, respectively, which are typical of PF resins.[S7]

Thermal and thermo-oxidative characterization of resins

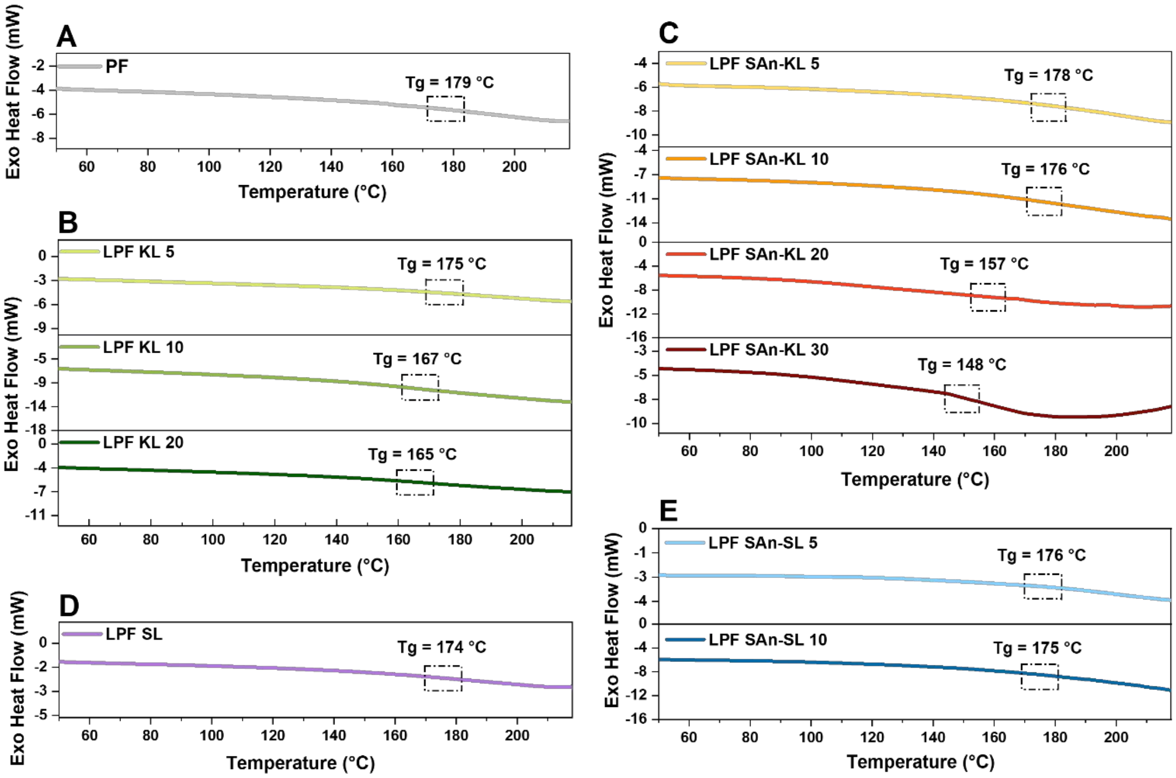

Figure S7 DSC of cured resins of **(A)** reference resin PF, **(B)** LPF based on pristine kraft lignin, **(C)** LPF based on succinylated kraft lignin, **(D)** LPF based on pristine soda lignin and **(E)** LPF based on succinylated soda lignin.

Table S2 Characteristic mass loss temperature related to 5% (T_5), 10% (T_{10}) and 50% (T_{50}) mass loss, maximum degradation rate temperature (DTG_{max}) and final residue at 750 °C (R_{750}) of reference resin PF, LPF based on pristine kraft lignin, LPF based on succinylated kraft lignin, LPF based on pristine soda lignin and LPF based on succinylated soda lignin.

Sample	T_5 (°C)	T_{10} (°C)	T_{50} (°C)	DTG_{max} (°C)	R_{750} (%)
PF	272	413	652	584	6.1
LPF KL 5	240	397	624	636	4.3
LPF KL 10	262	403	633	603	13.2
LPF KL 20	239	374	637	605	15.5
LPF SAn-KL 5	250	407	651	680	15.2
LPF SAn-KL 10	231	382	651	683	16.4
LPF SAn-KL 20	219	347	638	661	9.1
LPF SAn-KL 30	210	344	626	652	6.1
LPF SL 5	231	382	607	598	0.5
LPF SAn-SL 5	244	387	639	678	13.0
LPF SAn-SL 10	236	379	635	635	16.0

Mechanical behaviour of resins

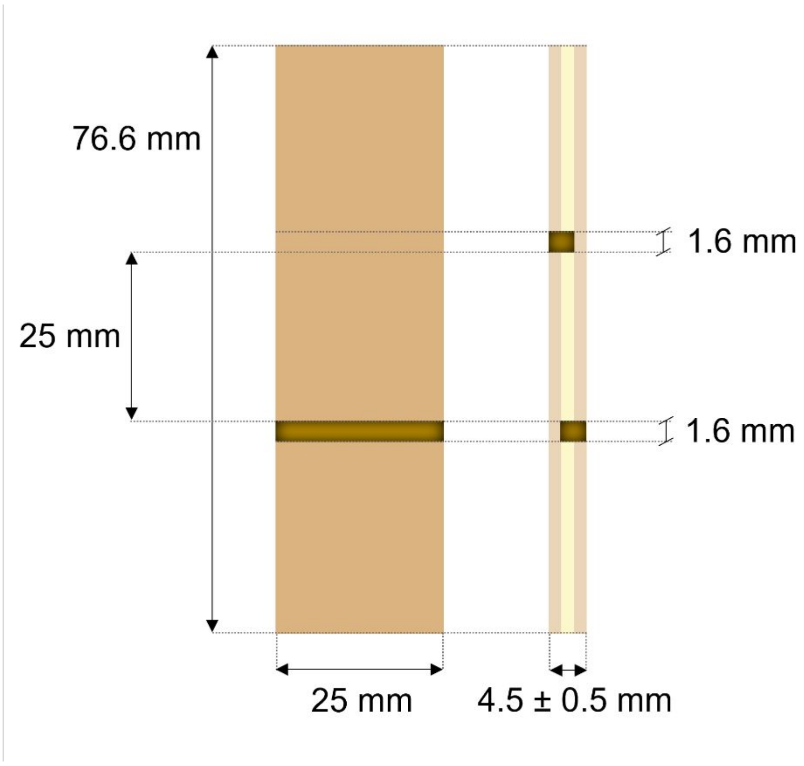

Figure S8 Geometry used for wood joint adhesion test.

The adhesive strength of wood-to-wood joint was tested in accordance with normative BS ISO 6237:2003. 1 g of resin blend was homogeneously spread on each wood layer. The wood sheets were overlayed paying close attention to the direction of the fibers, in such a way that the middle panel fibers were oriented orthogonally to the outer ones. After the curing step, panels were notched according to the normative, as schematized in Figure S8. The specimens as obtained were then loaded in tension on a Zwick-Roell dynamometer. Loading was applied at a uniformly increasing rate of 2.5 kN/min, hence a crosshead speed of 0.5 mm/min. The failure of a plywood can generally occur for either wood or adhesive failure. In the event of exposed adhesive after failure, a percentage assessment of the visible adhesive can be made. At least five testes for each sample were performed.

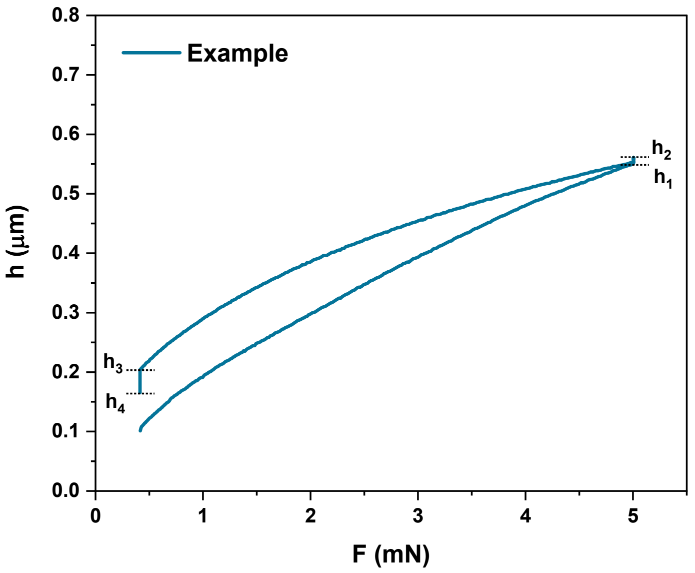

Figure S9 Example of typical microindentation cycle (penetration depth as function of applied load). Relevant penetration depths used for the calculations are marked.

- **η_{IT}**: amount of elastic work done by micro-indenting the sample, computed as the ratio between the elastic deformation work and the total deformation work performed during indentation, expressed in %.

- **$C_{IT,1}$**: Indentation creep, expressed in %. It describes the material behaviour at a constant maximum test load, calculated as indicated by Equation S1:

$$C_{IT,1} = \frac{h_2 - h_1}{h_1} * 100 \#(S1)$$

- **$C_{IT,2}$**: Recovery creep, expressed in %. It describes the material behaviour at a constant minimum test load after load reduction, calculated as expressed by Equation S2:

$$C_{IT,2} = \frac{h_4 - h_3}{h_3} * 100 \#(S2)$$

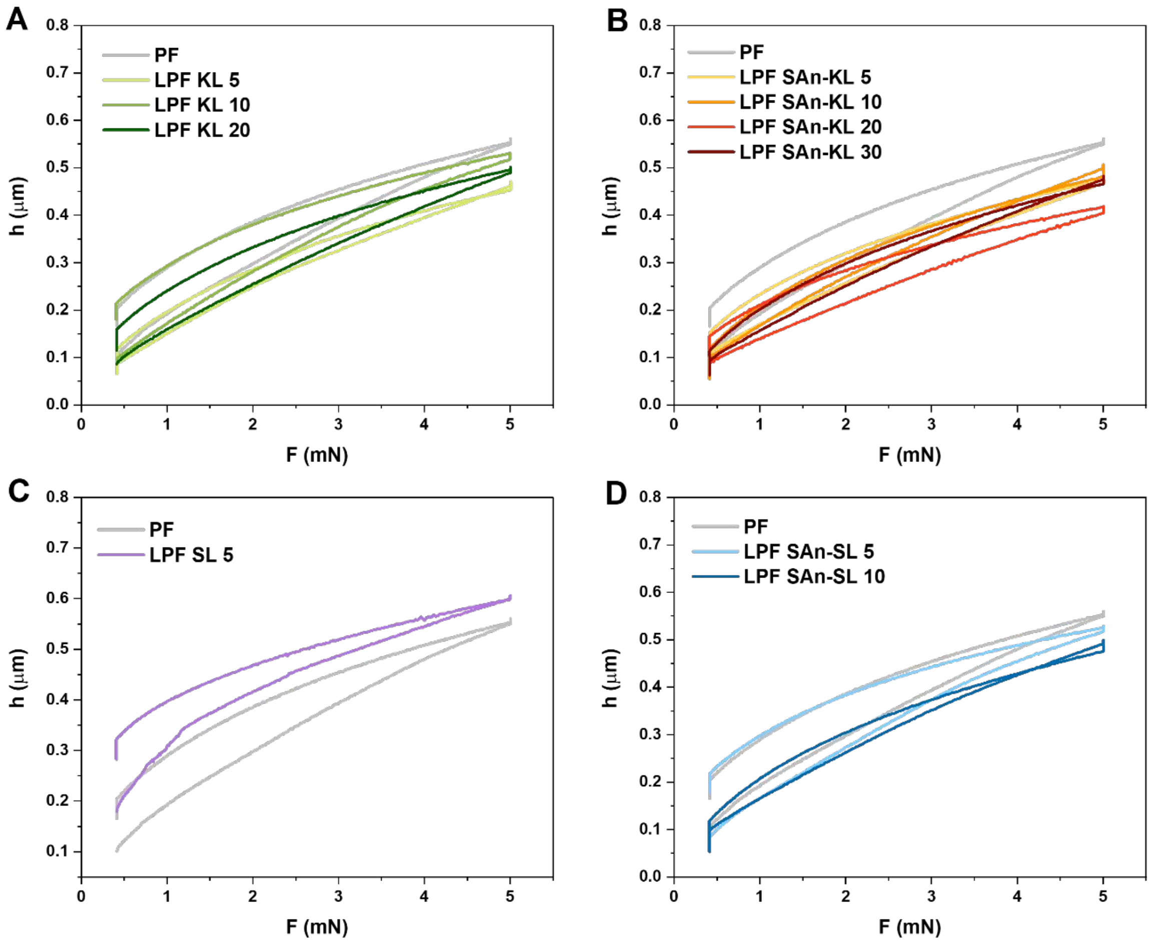

Figure S10 Representative example of microindentation cycle where penetration depth (h) is represented as a function of applied load (F) of **(A)** pristine kraft lignin-based resins, **(B)** succinylated kraft lignin-based resins, **(C)** pristine soda lignin-based resins and **(D)** succinylated soda lignin-based resins.

Microstructure of the cured resins

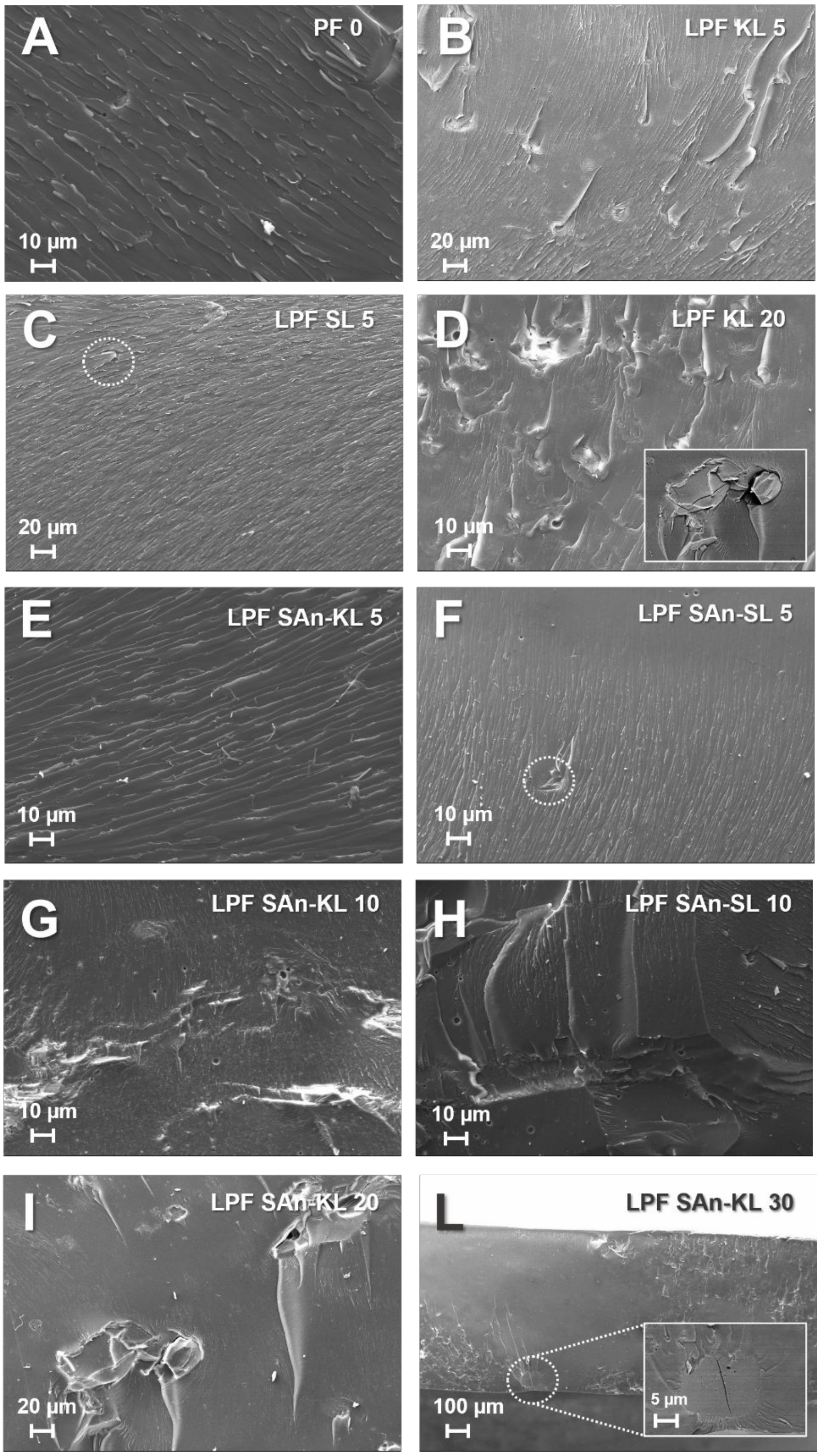

Figure S11 SEM images of PF and of some representative LPF resins.

SEM analyses were performed on all cured systems with the aim of studying their microstructure and morphology and to assess potential effects related to the introduction of different types and contents of lignin in the resins. The reference PF resin (Figure S11A) exhibits a regular and ladder-likely surface, with no evidence of surface defects or porosity, thus indicating efficient crosslinking and phase homogeneity. A sufficiently homogeneous surface is observed also in case of LPF KL 5 and LPF SL 5 (Figure S11B and S11C, respectively). A few lignin particles are clearly distinguishable in these samples, which are responsible for making the section rougher, likely contributing to initiate brittle fracture. Also in these cases, the surface does not present any kind of porosity, validating the suitability of the crosslinking process adopted. At higher pristine lignin substitution rate (i.e., 20 wt%, Figure S11D), the appearance of the resin cross-section is quite different. More kraft lignin particles become clearly visible within the resin matrix, as a result of their high concentration in the thermoset.

Considering the images related to modified lignin-based resins, similar remarks can be done. Indeed, at lower lignin substitution rate (i.e., LPF SAn-KL 5 and LPF SAn-SL 5 in Figure S11E and S11F, respectively), the fracture surface appears more regular and homogeneous, and no micro- or macro- porosity can be noted. Additionally, lignin particles are barely distinguishable, as a result of their excellent distribution and high incorporation level within the matrix. Conversely, approaching 10 wt% of lignin content in the resin, agglomerates of lignin particles are visible (Figure S11G and S11H) as well as some micro-porosities, as a possible consequence of lignin dehydration and/or presence of trapped volatile reaction byproducts (i.e., water vapour and ammonia). The agglomeration effect appears to be more pronounced when lignin is present at 20 or 30 wt% in the matrix (Figure S11I and S11L). Moreover, in the case of LPF SAn-KL 20, detachment of the lignin particles from the matrix is also observed, while in LPF SAn-KL 30 some microfractures originating from lignin particles embedded in the rigid matrix appear on the surface (highlighted with a dashed white circle in the micrographs).

References

(S1) Cateto, C. A.; Barreiro, M.F.; Rodrigues, A. E.;Brochier-Salon, M. C.; Thielmans, W.; Belgacem, M. N. Lignins as macromonomers for polyurethane synthesis: A comparative study on hydroxyl group determination, *Journal of Applied Polymer Science* **2008**, 109, 3008-3017.

(S2) He, G.; Riedl, B. Curing Kinetics of Phenol Formaldehyde Resin and Wood-Resin Interactions in the Presence of Wood Substrates. *Wood Science and Technology* **2004**, *38*, 69–81.

(S3) Rodrigues, J. S.; de Freitas, A. de S. M.; Maciel, C. C.; Mendes, S. F.; Diment, D.; Balakshin, M.; Botaro, V. R. Selection of Kraft Lignin Fractions as a Partial Substitute for Phenol in Synthesis of Phenolic Resins: Structure-Property Correlation. *Industrial Crops and Products* **2023**, *191*.

(S4) Poljanšek, I.; Krajnc, M. Characterization of Phenol-Formaldehyde Prepolymer Resins by in Line FT-IR Spectroscopy. *Acta Chimica Slovenica* **2005**, *52*, 238–244.

(S5) Tian, Y.; Huang, H.; Xu, X. Optimization of the Curing Process of Phenolic Impregnated Carbon Ablator. *Journal of Applied Polymer Science* **2018**, *135*, 46230.

(S6) Van Doorn, J.; Frijns, J. H. G.; Meijboom, N. Formation and reactions of bis(phosphino)succinic anhydrides. *Journal of the Chemical Society* **1990**.

(S7) Song, S.; Xu, Z.; Zhen, X.; Wang, Z.; Ge, T. Preparation of Lignosulfonate-Based Phenol Formaldehyde Foam with Excellent Thermal Performance. *Macromolecular Chemistry and Physics* **2022**, *223*, 1–10.
